# Tangshen Formula Improves Diabetes-Associated Myocardial Fibrosis by Inhibiting TGF-β/Smads and Wnt/β-Catenin Pathways

**DOI:** 10.3389/fmed.2021.732042

**Published:** 2021-12-06

**Authors:** Lin Hu, Yuyang Wang, Yuzhou Wan, Liang Ma, Tingting Zhao, Ping Li

**Affiliations:** ^1^Beijing Key Laboratory for Immune-Mediated Inflammatory Diseases, Institute of Clinical Medical Sciences, China-Japan Friendship Hospital, Beijing, China; ^2^National Energy R&D Center for Biorefinery, Beijing University of Chemical Technology, Beijing, China; ^3^College of Pharmacy, Weifang Medical University, Weifang, China

**Keywords:** Tangshen Formula, myocardial fibrosis, TGF-β/Smad, Wnt/β-catenin, KKAy

## Abstract

Cardiovascular disease has become the main cause of death among complications of diabetes. Myocardial fibrosis is a crucial pathological change of cardiovascular disease. Tangshen Formula (TSF) shows a good clinical effect in the treatment of diabetic kidney disease (DKD). However, whether TSF alleviates diabetes-associated myocardial fibrosis is still unknown. In the present research, we studied the effect and mechanism of TSF in the treatment of myocardial fibrosis *in vivo* and *in vitro*. We found that TSF treatment significantly downregulates myocardial fibrosis-related markers, including collagens I and III, and α-SMA. TSF also protects primary mouse cardiac fibroblast (CF) from transforming growth factor-β1- (TGF-β1-) induced damage. Moreover, TSF decreased the expression levels of TGF-β/Smad-related genes (α-SMA, collagens I and III, TGF-β1, and pSmad2/3), and increased Smad7 gene expression. Finally, TSF decreased the expressions of wnt1, active-β-catenin, FN, and MMP7 to regulate the Wnt/β-catenin pathway. Taken together, TSF seems to attenuate myocardial fibrosis in KKAy mice by inhibiting TGF-β/Smad2/3 and Wnt/β-catenin signaling pathways.

## Introduction

Diabetes mellitus (DM) is a global epidemic. Nearly half a billion people worldwide have diabetes, and this number is expected to increase by 25% in 2030 and by 51% in 2045 (1). Among the complications of diabetes, diabetic cardiomyopathy is a common cause of death (2). Myocardial fibrosis is a typical pathology in cardiovascular diseases and is marked by profuse deposition and disruption of extracellular matrix (ECM) and the over-proliferation of activated cardiac fibroblasts (CFs) (3). Despite strict blood glucose control, the incidence of diabetic cardiomyopathy remains high. Thus, it is crucial to search for new drugs to treat myocardial fibrosis.

Tangshen Formula (TSF) is a Chinese herbal medicine (CHM) for the treatment of diabetic kidney disease (DKD) (4). In both rat and mouse models of DKD, TSF has been found to effectively treat renal fibrosis by inhibiting the transforming growth factor β (TGF-β)/Smad signaling pathway (5). It is well known that TGF-β/Smad signaling is a key pathway in the development of fibrosis in many organs, including the heart (6). Also, TGF-β1 is proved to play a critical pathogenic role in diabetes-associated myocardial fibrosis. It was potentially found that TGF-β1 could induce the expression of ECM protein in CFs by activating Smads-dependent signals in diabetic mice, thus leading to pathological fibrosis (7). TGF-β1 binds to its receptor and activates downstream mediators, including Smad2 and Smad3, to exert biologic effects; TGF-β1 is negatively regulated by Smad7 expression. The overexpression of TGF-β1 causes the overproduction of ECM protein and inhibits their degradation, leading to fibrosis (6). Ho et al. reported that TGF-β1 cooperates with Wnt protein signaling to control biologic activities in a variety of cells (8). The Wnt signaling pathway comprises two highly conserved pathways, among which the canonical β-catenin-dependent pathway is involved in myocardial fibrosis (9). β-catenin forms a complex in the nucleus with transcriptional factors of T-cell factor/lymphoid enhancer factor (TCF/LEF) to stimulate the transcription of Wnt target genes, thereby resulting in the deposition of ECM (10).

In this study, we aimed to study the therapeutic effect of TSF on myocardial fibrosis and its underlying mechanism. We found that TSF can alleviate myocardial fibrosis in KKAy mice and fibrotic injury in TGF-β-stimulated myocardial fibroblasts by inhibiting the TGF-β/Smad pathway and the Wnt/β-catenin pathway. This study provided both *in vivo* and *in vitro* evidence for the potential application of TSF in the treatment of myocardial fibrosis.

## Materials and Methods

### Herbal Formulation

Tangshen Formula is made up of seven kinds of herbs comprising astragalus root, burning bush twig, rehmannia root, bitter orange, cornus fruit, rhubarb root and rhizome, and notoginseng root. It was prepared by Beijing Yadong Biopharmaceutical (Beijing, China), and the standardization of the formula was in accordance with the *Pharmacopoeia of The People's Republic of China 2015* (11).

### Animals and Experimental Design

Eighteen diabetic male KKAy mice (8 weeks old, 20–25 g) and six male C57BL/6J mice were purchased from Beijing Vital River Laboratory Animal Technology (Beijing, China). All animals were housed at 23 ± 3°C and 55 ± 15% humidity with 12:12 h light/dark cycle and were allowed access to chow and water *ad libitum*. KKAy mice were treated with a high-fat diet, and their blood glucose was measured once in 2 weeks by One Touch Ultra blood glucose monitoring system (LifeScan, Milpitas, CA, USA). The KKAy mice were randomly divided into the following three groups: (1) KKAy group: KKAy mice-administered distilled water (*n* = 6); (2) KKAy+TSF group: KKAy mice-administered TSF (3.57 g/kg per day) by oral gavage (*n* = 6) (12); and (3) KKAy+Irbesartan group: KKAy mice-administered irbesartan by oral gavage (22.5 mg/kg per day) (*n* = 6). The control group consisted of healthy C57BL/6J mice-administered distilled water (*n* = 6).

All the animals were weighed once a week. After 16 weeks, all mice were sacrificed under anesthesia. Serum was collected for assay. The apex of the heart from each animal was used for a pathologic analysis, and the rest of the heart tissue was used for a molecular biologic analysis. Animal care and treatments were in accordance with the NIH Guiding Principles for the Care and Use of Laboratory Animals, and the protocol was approved by the Beijing National Proteome Science Center Animal Management Ethics Committee.

### Heart Histology and Immunohistochemistry

The apex of the heart tissue was fixed with 4% phosphate buffered saline (PBS) buffered paraformaldehyde, embedded in paraffin, and then sectioned into 3 μm thicknesses and stained with Masson trichrome according to the standard procedure. Masson trichrome staining causes muscle fibers to turn red and collagen fibers to turn blue. Immunohistochemistry (IHC) staining was used to detect the distribution and expression of biomarkers for fibrosis. We used a microwave-based heating antigen retrieval and 3% H_2_O_2_ to block non-specific staining. The antibodies used included: collagen I (1310-01, SouthernBiotech, Birmingham, AL, USA; 1:200 dilution), collagen III (GB111629, Servicebio, Wuhan, China; 1:200 dilution), α-SMA (GM085102, Gene Tech, Shanghai, China; 1:200 dilution), phosphorylated Smad2/3 (sc-11769, Santa Cruz Biotechnology, Dallas, TX, USA; 1:200 dilution), and anti-β-catenin (610154, BD Transduction Laboratories, Shanghai, China; 1:50 dilution). Images were quantitatively analyzed using Image-Pro Plus v 6.0 (Media Cybernetics, Rockville, MD, USA). Briefly, the accumulation of collagens I and III and α-SMA in 10 random areas, except staining around blood vessels, was analyzed under 200× for the percentage of positive areas in the examined field. Numbers of p-Smad2/3+ cells were counted in 10 random areas for each sample under 400×.

### Ultrastructure of Myocardium

Heart ultrastructure was detected using transmission electron microscopy (JEOL-100CXII; JEOL, Tokyo, Japan). The heart was sectioned into 1 mm cubes and fixed with 2.5% glutaraldehyde at 4°C for 24 h, and then embedded in an epoxy resin. Ultrathin sections were sliced and stained with uranyl acetate and lead citrate at room temperature, for 30 and 15 min, respectively. Twenty images per sample were randomly selected and observed, and the ultramicrostructure of the myocardium was observed under the magnification ×12,000.

### Isolation of Primary Mice Myocardial Fibroblasts

Cardiac fibroblasts were derived from the hearts of neonatal mice. The hearts were removed from the C57BL/6J newborn mice (Beijing Vital River Laboratory Animal Technology, Beijing, China) and, under aseptic conditions, were sectioned into tissue blocks of about 1 mm^3^. The tissue blocks were washed with Hanks Balanced Salt solution without Ca^2+^ and Mg^2+^ and digested at 37°C with 0.08% trypsin and 0.06% collagenase. The digested supernatant was collected and neutralized with low glucose (5 mmoL/L) Dulbecco Modified Eagle medium/fatty acid (DMEM/FA) containing 20% serum until the tissue mass became transparent and digestion was terminated. The resulting suspension was filtered with a 200-μm BD Falcon cell strainer (Corning Life Sciences, Corning, NY, USA) and centrifuged from 4 to 10 min at 25–300 × g. The filtered solution was then plated onto culture dish.

### Cell Viability

Primary mice myocardial fibroblasts were cultured in DMEM with 10% fetal bovine serum (GIBCO, Carlsbad, CA, USA) at 37°C with 5% CO_2_. To investigate the role of TGF-β1, the fibroblasts were exposed to TGF-β1 for 6, 16, 24, 36, and 72 h sequentially at 0, 2.5, 5, 10, and 15 μg/ml, respectively. After the TGF-β1-stimulated myocardial fibroblast injury model was successfully obtained, the cells were treated with TSF for 72 h at the concentrations of 100 and 250 μg/ml.

An MTT assay was used to detect whether TSF had an effect on cell viability. Being starved overnight with DMEM/F12 medium without fetal bovine serum (FBS), C57BL/6J mouse primary myocardial fibroblasts were then grown in 96-well plates and incubated with TSF at the concentrations of 0, 100, 250, 500, 750, and 1,000 μg/ml for 72 h. Each well was added with 1/10 volume of MTT solution and incubated at 37°C with 5% CO_2_ for 4 h. After adding formazan lysate, the assay was performed at 490 nm wavelength with a microplate analyzer (Thermo Fisher Scientific, Waltham, MA, USA). The experimental methods were completed according to the kit instructions, and cell viability was calculated.

### Real-Time Quantitative PCR

Total RNA was isolated from mouse hearts using the TriZol method. RNA concentration was then quantified using a NanoDrop-1000 spectrophotometer (NanoDrop Technologies, Wilmington, DE, USA). Total RNA was reverse transcriptased to the complementary DNA (cDNA) template using the M5 SuperFast qPCR RT kit (Ju Hemei, Beijing, China) quantitative PCR (qPCR) was performed with the QuantStudio 5 Real-Time PCR system (Applied Biosystems, Waltham, MA, USA) using the IQ SYBR Green Supermix reagent (CWBIO, Beijing, China), as previously described (13). Glyceraldehyde-3-phosphate dehydrogenase (GAPDH) was used as the internal control to normalize the gene expression in the same cDNA. This was followed by applying the ΔΔ*C*(*t*) threshold cycle method, to analyze the qPCR data (14). The specific primer sequences used are listed in Table 1.

**Table 1:** List of primers used for quantificational real-time polymerase chain reaction (qRT-PCR).

**Gene**	**Forward primer**	**Reverse primer**	**PCR size (bp)**
GAPDH	TGTTTCCTCGTCCCGTAGA	ATCTCCACTTTGCCACTGC	106
TGF-β1	TGGGGACTTCTTGGCACT	ATAGGGGCGTCTGAGGAAC	117
α-SMA	TACTGCCGAGCGTGAGA	TCCAGGGAGGAAGAGGAG	105
ColI	CAGAGGCGAAGGCAACA	GTCCAAGGGAGCCACATC	145

### Western Blot Analysis

Mouse heart tissues were homogenized in a mixture of radioimmunoprecipitation assay (RIPA) buffer and 1% cocktail (Bimake, Houston, TX, USA) to extract the protein. Protein concentration was measured using the Bicinchoninic acid assay (BCA) Protein Assay kit (Epizyme Biotech, Shanghai, China) and then was denatured. The proteins were separated by 12% sodium dodecyl sulfate (SDS) and transferred to polyvinylidene difluoride (PVDF) membranes. After blocking in 5% skim milk for 1 h, the membranes were incubated with primary antibody at 4°C overnight. The following day, the membranes were washed three times with tris-buffered saline and Tween-20 (TBST) and incubated with secondary antibody. Membrane immunostaining was observed and analyzed using the ChemiDoc XRS system (Bio-Rad, Hercules, CA, USA). Signals were quantified by the Image J program (National Institutes of Health, Bethesda, MD, USA). β-actin normalization detected the protein ratio and was expressed as mean ± SME.

The primary and secondary antibodies were: TGF-β1 (1:1,000 dilution; BS1361, Bioworld Technology, Nanjing, China), smad7 (1:500 dilution; sc-9183, Santa Cruz Biotechnology), anti-rabbit (1:5,000 dilution; S8002, Beijing Guanxingyun Sci and Tech, Beijing, China), anti-mouse (1:5,000 dilution; S8001, Beijing Guanxingyun Sci and Tech, Beijing, China), anti-goat (1:2,000 dilution; ab205723, abcam, Cambridge, UK), FN (1:500 dilution; sc-8422, Santa Cruz Biotechnology), wnt1 (1:1,000 dilution; AF5315, Affinity Biosciences, Cincinnati, OH, USA), β-catenin (1:500 dilution; sc-7963, Santa Cruz Biotechnology), active-β-catenin (1:500 dilution; 05-665, Merck Millipore, Darmstadt, Germany), and MMP7 (1:1,000 dilution; ER1913-08, Huabio, Woburn, MA, USA).

### Statistical Analysis

All data collected from this study were expressed as means ± SEM. The statistical analysis was performed and viewed with the GraphPad Prism software (LaJolla, CA, USA), and differences among the means were assessed using a one-way ANOVA.

## Results

### Effect of TSF on Body Weight and Blood Glucose

**Figure 1:**
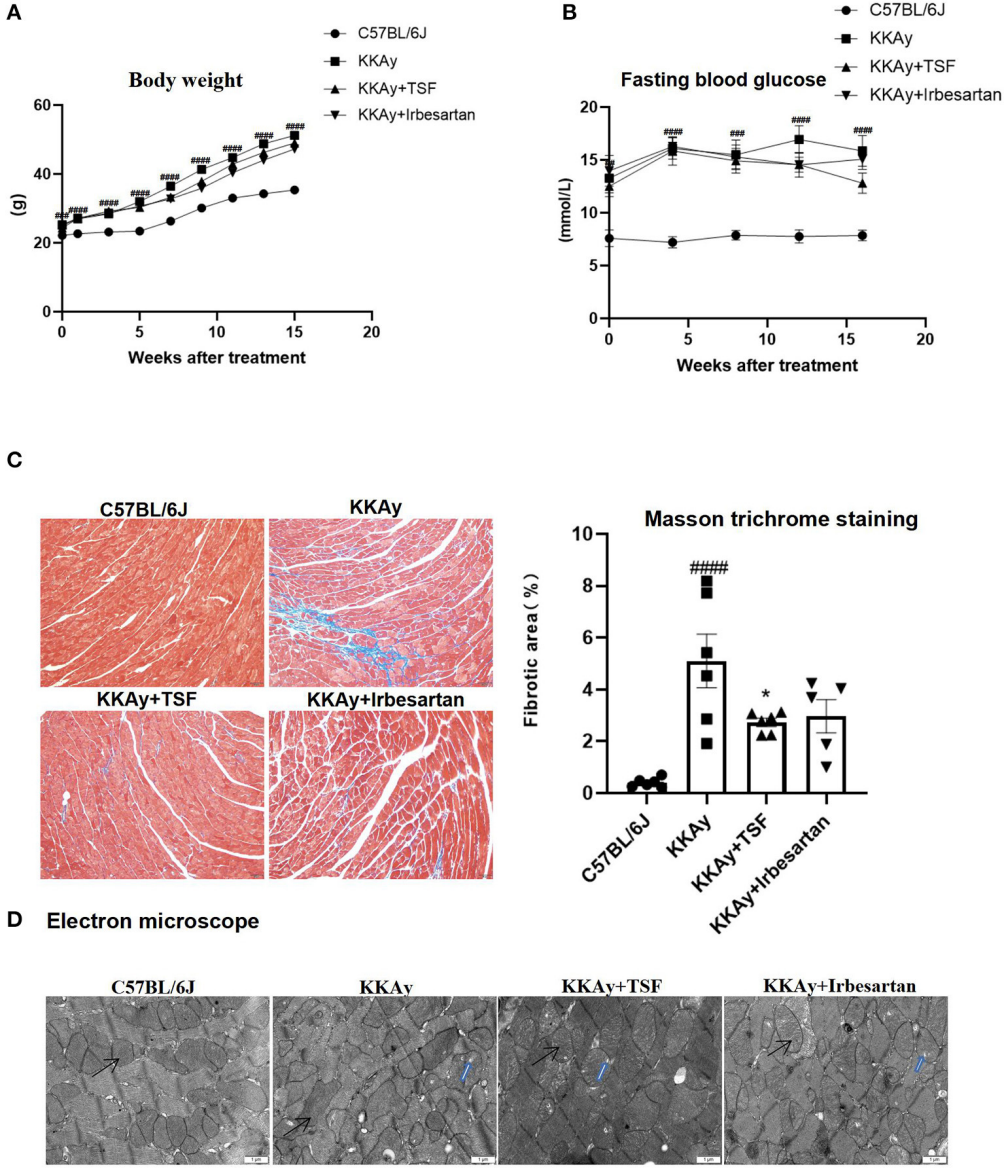
Pathologic changes after Tangshen Formula (TSF) and irbesartan treatments. **(A)** Body weights were recorded once a week. Data are expressed as mean ± SEM. ^###^*p* < 0.001, ^####^*p* < 0.0001 KKAy group vs. C57BL/6J group. **(B)** Fasting blood glucose. After 16 weeks, glucose levels in KKAy mice were significantly increased. TSF-treated KKAy mice exhibited no statistical differences in glucose levels. Data are expressed as mean ± SEM. ^###^*p* < 0.001, ^####^*p* < 0.0001 KKAy group vs. C57BL/6J group. **(C)** Histology (Masson trichrome staining, original magnification, 200×) and semiquantification of the collagen area. Data are expressed as mean ± SEM. *n* = 6. *p < 0.05 TSF-treated group vs. KKAy group; ^###^*p* < 0.001 ^####^*p* < 0.0001 KKAy group vs. C57BL/6J group. **(D)** Ultrastructural findings of myocardial tissues in four groups of mice. Magnification ×12,000. Scale bar = 1 μm. Black arrows indicate mitochondria. Blue arrows indicate mitochondria vacuolation.

During the 16 weeks of treatment with TSF or irbesartan, there were no deaths observed in KKAy mice. Body weights of all animals were measured once a week, and weight gain was recorded (Figure 1A). Weight was significantly increased in the KKAy group compared to the C57BL/6J group. However, in the treatment groups, weights were significantly decreased due to TSF or irbesartan therapy. After 16 weeks of treatment, blood glucose levels in KKAy+TSF and KKAy+irbesartan groups were lower than the levels in the KKAy group, but without a significant difference (Figure 1B).

Irbesartan ameliorates myocardial fibrosis in diabetic cardiomyopathy (15). It is an angiotensin domain receptor inhibitor and is considered to be a common medication for improving the structure and function of cardiomyocytes and reducing the risk of death from heart failure (16). Therefore, irbesartan was used as a positive control group to investigate the effects of drug therapy.

### TSF Treatment Attenuates Cardiac Injury in Diabetic Mice

Masson trichrome staining revealed a large number of blue collagen fibers in the KKAy group compared with the C57BL/6J group. In contrast, in TSF and irbesartan groups, the blue collagen area was significantly reduced (Figure 1C). Moreover, quantitative data demonstrated that the treatment with TSF for 16 weeks significantly attenuates these histologic injuries.

Under electron microscopy, the ultrastructure of myocardial cells was observed and the mitochondria remained intact in the C57BL/6J group. In the KKAy group, the myocardial structure was diminished and mitochondrial vacuolization and crest dissolution were evident. In the KKAy+TSF group, the myocardial ultrastructural injury was alleviated though mitochondria did exhibit slight vacuolization. There were no obvious pathologic changes in the KKAy+TSF and KKAy+ irbesartan group compared with the C57BL/6J group (Figure 1D).

### TSF Inhibited Myocardial Fibrosis in Diabetic Mice

**Figure 2:**
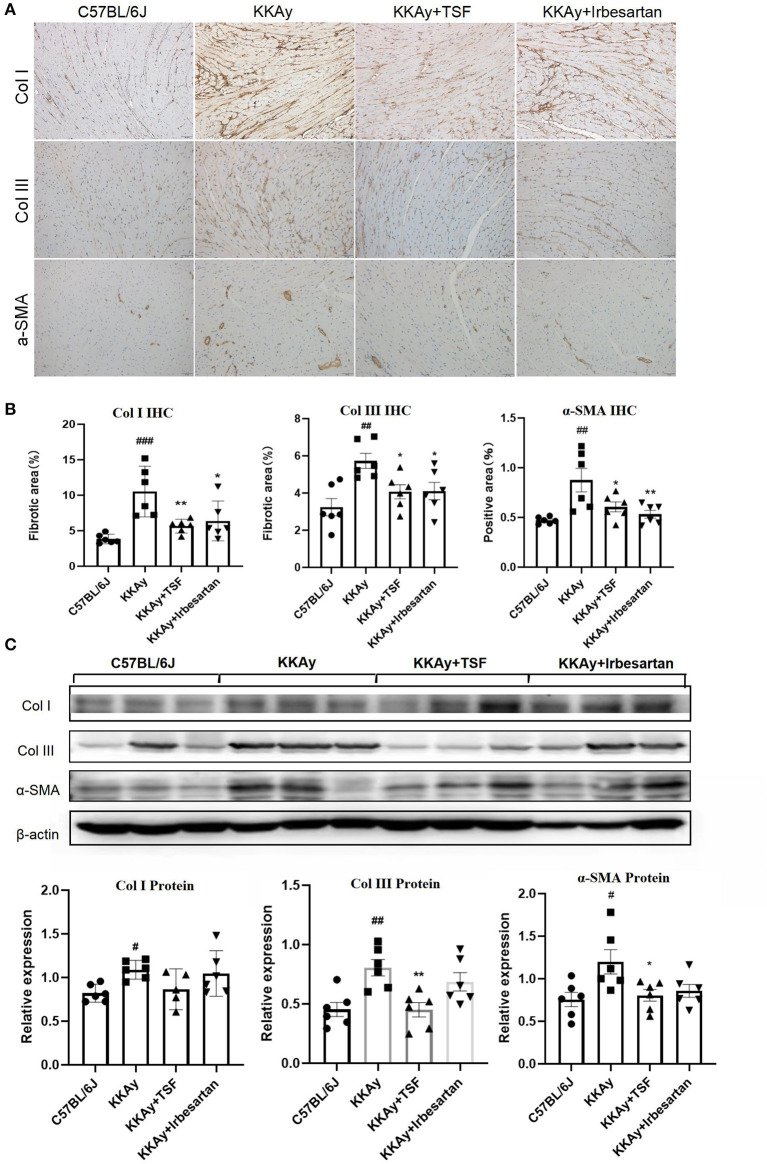
Tangshen Formula inhibited myocardial fibrosis in KKAy mice. **(A)** Immunohistochemistry (IHC) of collagen III (ColIII, original magnification, 200×), collagen I (ColI, original magnification, 200×), and alpha smooth muscle actin (α-SMA, original magnification, 200×). **(B)** IHC and semi-quantitative analyses for collagens I and III, and α-SMA. Data are expressed as mean ± SEM (*n* = 6). *p < 0.05, **p < 0.01 TSF-treated group vs. KKAy group; ^#^*p* < 0.05, ^##^*p* < 0.01, ^###^*p* < 0.001 KKAy group vs. C57BL/6J group. **(C)** Western blot and semi-quantitative analyses of ColI, ColIII, and α-SMA expressions.

One of the main symptoms of myocardial fibrosis is the accumulation of collagen in the ECM. Collagen I account for about 80% of the collagen in the heart muscle and increases in myocardial fibrosis. Collagen III is also an important component of the cardiac ECM (17). α-SMA can be assembled into stress fibers to reshape the surrounding ECM. Thus, in our study, we examined the therapeutic effect of TSF on myocardial fibrosis in KKAy diabetic mice. In the heart samples, the immune-positive areas of collagens I and III and α-SMA were yellow-brown (Figure 2A). IHC revealed that KKAy mice developed myocardial fibrosis, including a significant upregulation of collagens I and III as well as α-SMA, which were attenuated by the treatment with TSF or irbesartan (Figure 2B). Similarly, western blotting revealed that the upregulation of collagens III and α-SMA were significantly decreased after TSF treatment (Figure 2C).

### TSF Ameliorated Myocardial Fibrosis by Controlling the TGF-β/Smad Pathway in KKAy Mice

**Figure 3:**
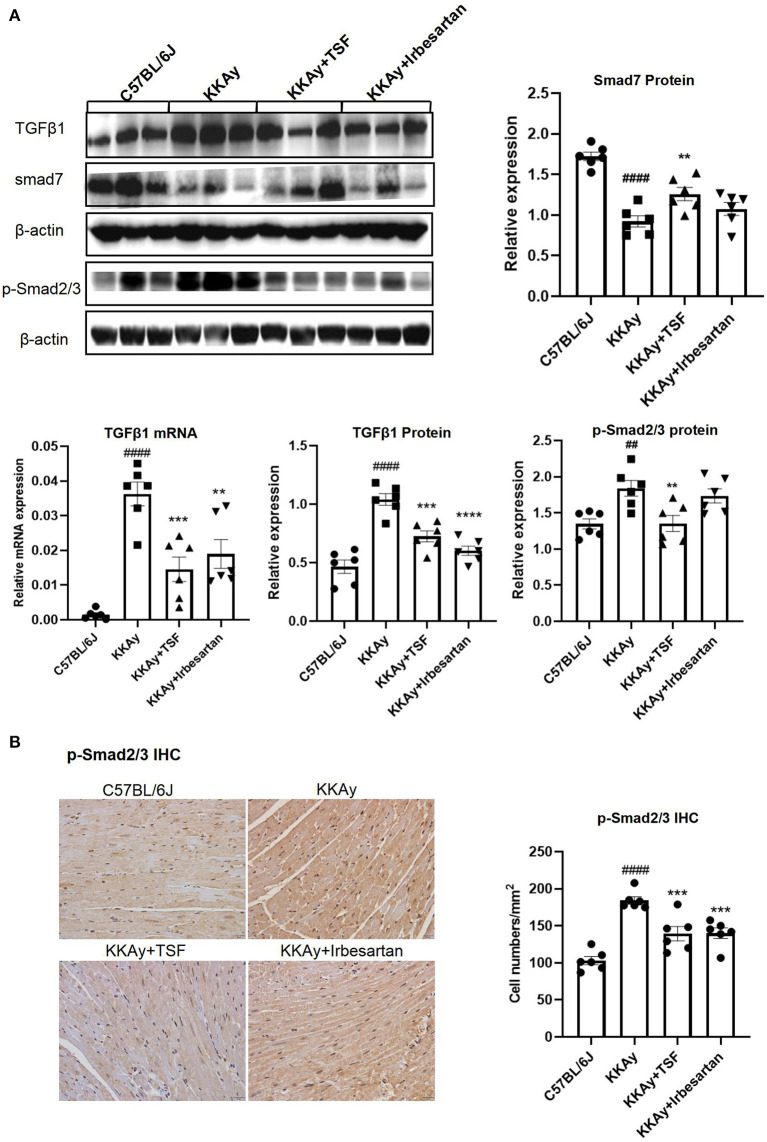
Tangshen Formula treatment inhibited the expression of transforming growth factor-β1 (TGF-β1) and p-Smad2/3 and promoted the expression of smad7 in KKAy mice. **(A)** Western blot and semi-quantitative analyses of TGF-β1, smad7, p-Smad2/3, and Smad2/3 expressions. **(B)** IHC of psmad2/3 (original magnification, 400×) and the number of positive cells in each group. Data are presented as mean ± SEM. **p < 0.01, ***p < 0.001, ****p < 0.0001 TSF-treated group vs. KKAy group; ^##^*p* < 0.01, ^####^*p* < 0.0001 KKAy group vs. C57BL/6J group.

Real-time qPCR and western blot revealed that KKAy mice developed myocardial fibrosis, including a significant upregulation of TGF-β1 at the messenger RNA (mRNA) and protein levels as well. This upregulation was inhibited after TSF treatment (Figure 3A). IHC results of p-Smad2/3 and western blot analyses of p-Smad2/3 showed that the activation of TGF-β/Smad2/3 signaling in the KKAy group and its effect were reduced after TSF treatment (Figure 3B). The protein expression level of smad7 was markedly decreased in KKAy mice compared with the C57BL/6J group (*p* < 0.01), whereas the protein expression was significantly increased in the TSF group compared with the KKAy group (Figure 3A). No significant difference was found between C57BL/6J and TSF groups. These results indicated that TSF is effective in inhibiting the TGF-β/Smad pathway in KKAy mice.

### Effect of TSF on Wnt/β-Catenin Signaling Pathway in KKAy Mice

**Figure 4:**
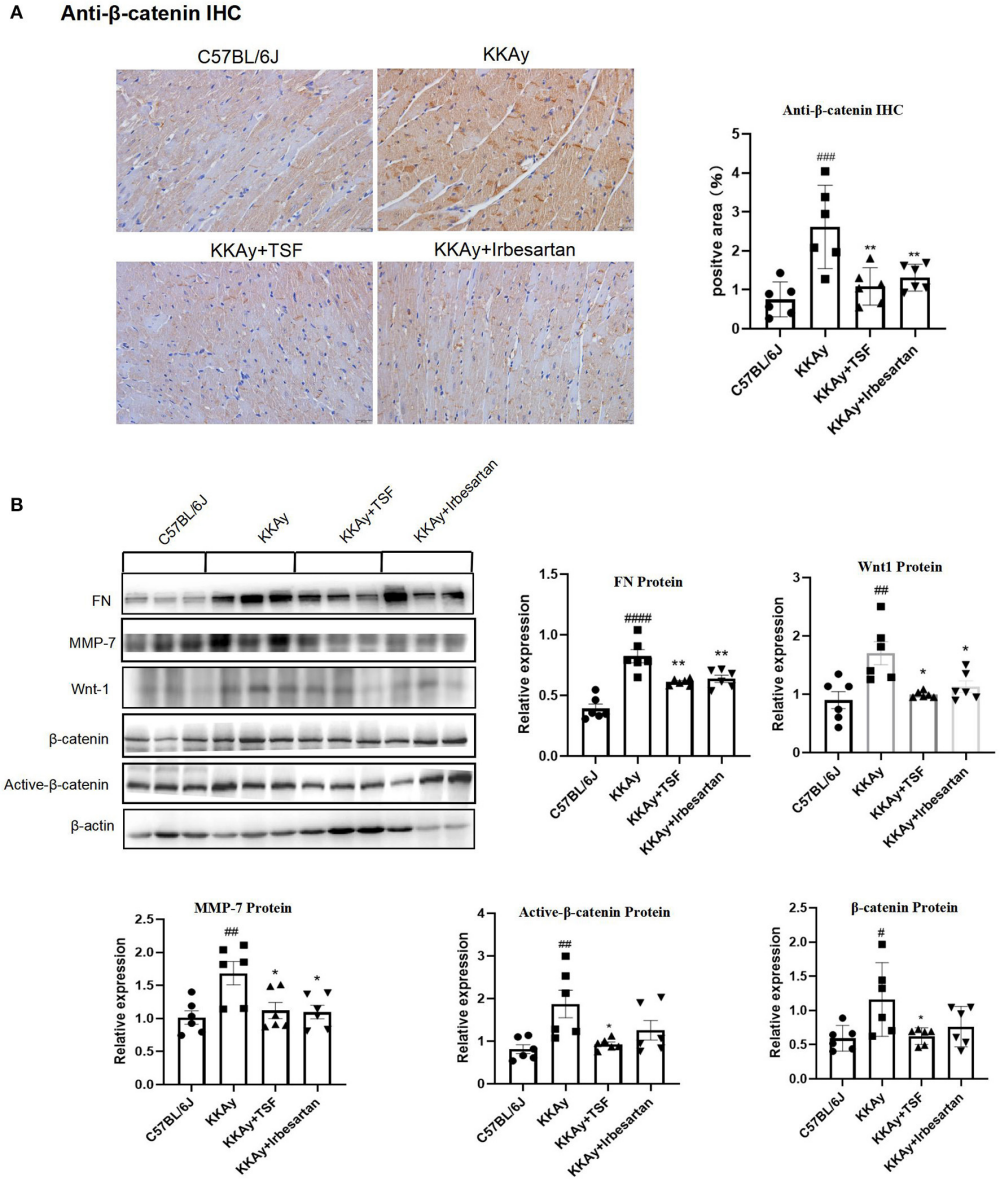
An effect of TSF on the canonical Wnt pathway. **(A)** IHC of anti-β-catenin (original magnification, 200×). Data are presented as mean ± SEM. *p < 0.05, **p < 0.01 TSF-treated group vs. KKAy group; ^#^*p* < 0.05, ^##^*p* < 0.01, ^###^*p* < 0.001 KKAy group vs. C57BL/6J group. **(B)** Western blot analyses and semi-quantitative analyses revealed expressions of Wnt1, active-β-catenin, β-catenin, FN, and MMP-7 in KKAy mice and the different treatment groups. ^#^*p* < 0.05, ^##^*p* < 0.01, ^###^*p* < 0.001, ^####^*p* < 0.0001 KKAy group vs. C57BL/6J group. *p < 0.05, **p < 0.01 TSF-treated group vs. KKAy group.

To confirm the effect of TSF on Wnt/β-catenin signaling, the western blot analysis was used to detect Wnt1 and β-catenin and their downstream target MMP7, FN. Immunohistochemical analysis of anti-β-catenin showed that the relative expression of anti-β-catenin was upregulated in the KKAy group and was significantly reduced after the treatment with TSF, which demonstrated the activation of Wnt/β-catenin (Figure 4A). The presence of the bands only at the target protein sites indicates that the staining is specific. The expression of Wnt1, β-catenin, and active-β-catenin was increased in the KKAy mice compared with the C57BL/6J group, demonstrating that the Wnt/β-catenin pathway was active in the KKAy animal model. TSF decreased the expression of Wnt1, β-catenin, and active-β-catenin proteins in the TSF treatment group compared with the KKAy group. Irbesartan also showed an inhibitory effect on Wnt1 and active-β-catenin expressions. A significant difference in the levels of MMP7 and FN expressions was found between the C57BL/6J group and KKAy group (*p* < 0.01) as well as between the KKAy group and TSF group. In the KKAy group, the expressions of MMP7 and FN significantly increased compared with the C57BL/6J group. In contrast, TSF treatment decreased the expression levels of MMP7 and FN (Figure 4B), suggesting that the treatment with TSF attenuates diabetic myocardial fibrosis *via* the Wnt/β-catenin mechanism. Taken together, this study highly suggests that TSF has an inhibitory effect on the Wnt/β-catenin signaling pathway.

### TSF Improved CFs Injury, Reduced Expressions of Collagens I and III as Well as α-SMA in C57BL/6J Mice CFs

**Figure 5:**
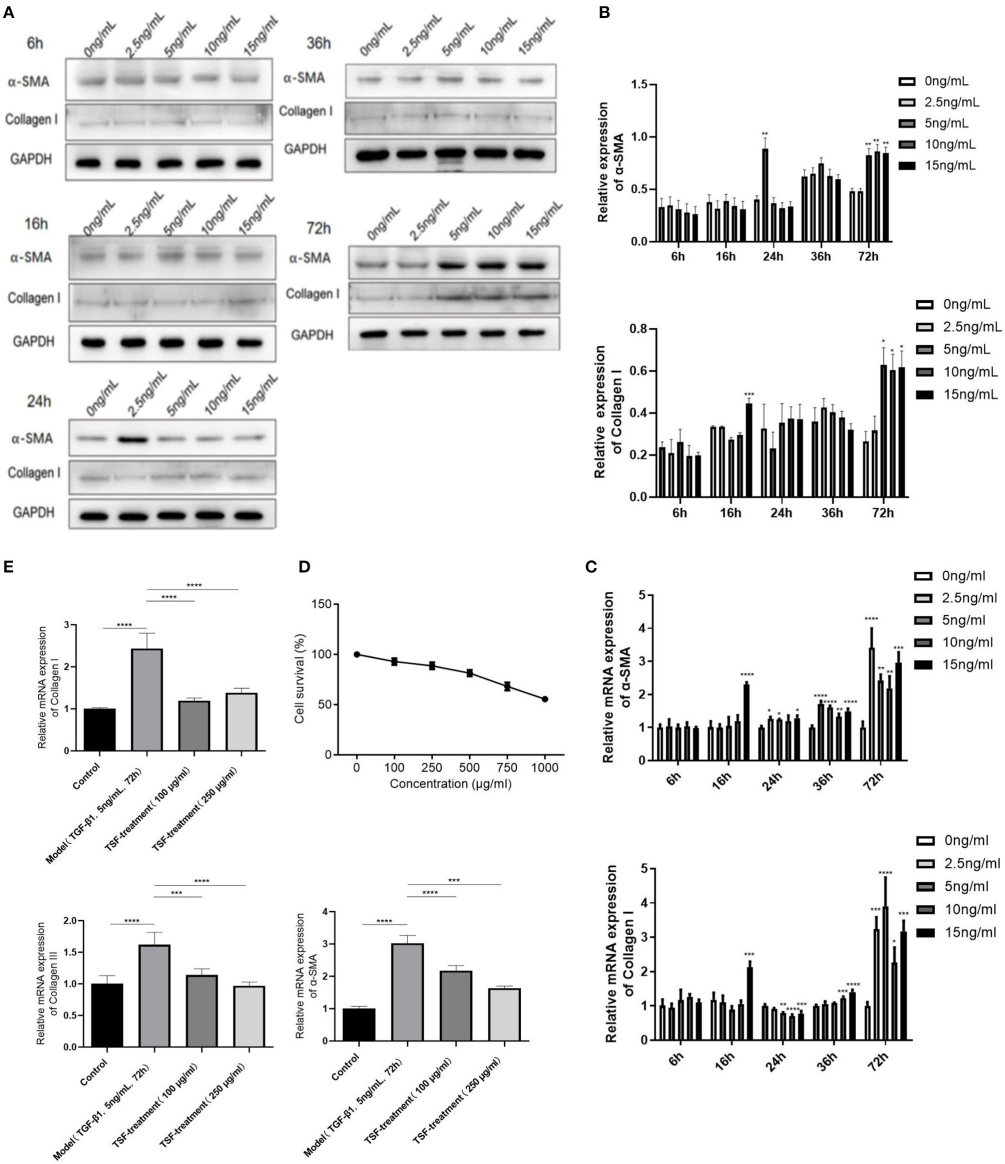
An inhibitory effect of TSF against the activation of the TGF-β/smad pathway *in vitro*. **(A)** The expression of fibrosis index protein in cardiac fibroblast (CF) cells stimulated by TGF-β in different concentrations at different times. **(B)** Quantitative analyses of protein expressions. **(C)** Quantificational real-time polymerase chain reaction (qRT-PCR) of messenger RNA (mRNA) expressions of fibrosis index of CFs induced by TGF-β1. **(D)** Effects of different concentrations of TSF on the survival rate of cells. **(E)** qRT-PCR of mRNA expressions of collagens I and III and α-SMA in CFs induced by TGF-β1 in the different groups. Data are presented as mean ± SEM. *p < 0.05, **p < 0.01, ***p < 0.001, ****p < 0.0001.

Combined with protein and mRNA expressions of α-SMA and collagen I, the optimal myocardial fibroblast fibrotic injury model was obtained under the condition of 5 ng/ml TGF-β1 induced for 72 h (Figures 5A–C). To determine whether TSF affected cell proliferation, MTT assays were used to investigate the cytotoxicity of TSF (Figure 5D). The results showed that the cell survival rate decreased gradually as TSF concentration increased. Thus, the concentrations of 100 and 250 μg/ml TSF were chosen for the *in vitro* experiments.

Cardiac fibroblasts induced by TGF-β1 showed an upregulation of mesenchymal markers, including collagens I and III, and α-SMA (Figure 5E), which were all significantly downregulated after 100- and 250-μg/ml TSF treatment.

## Discussion

**Figure 6:**
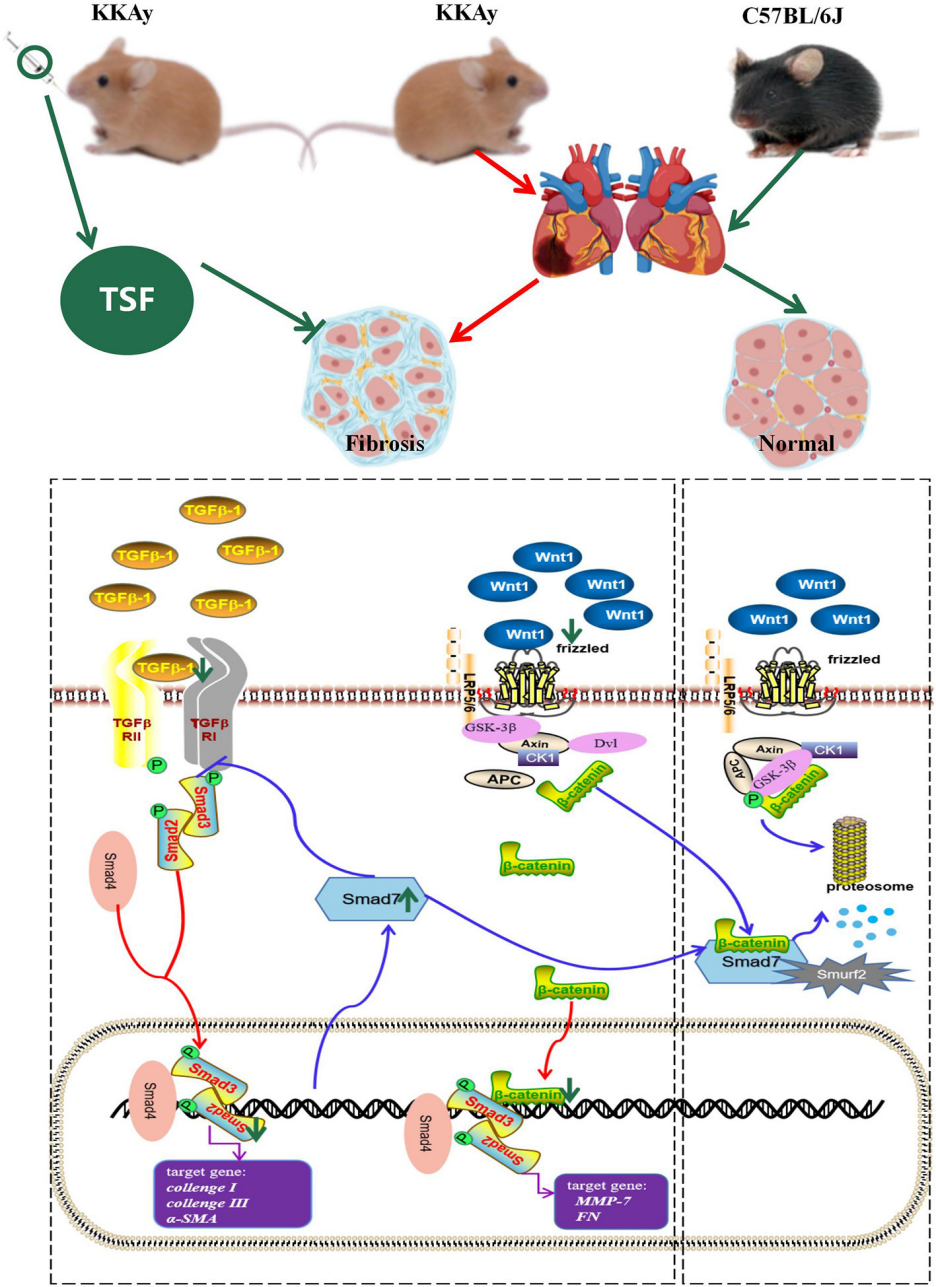
Schema of the possible mechanism of antifibrosis action of TSF. In the TGF-β/smad pathway, TSF decreased the expression of TGF-β1, inhibited the phosphorylation of Smad2/3, and increased the expression of Smad7, which promoted the degradation of catenin. Meanwhile, TSF also inhibited the Wnt pathway and further promoted β-catenin phosphorylation and degradation. The green arrow represents the effect of TSF.

Our study provides evidence that the Chinese traditional herbal medicine TSF markedly alleviated myocardial fibrosis in KKAy mice. The therapeutic impact of TSF on diabetes-associated myocardial fibrosis was related to the inhibitory effect of TGF-β/Smad and Wnt/β-catenin-mediated myocardial fibrosis (Figure 6).

In this study, we validated that TSF could relieve fibrosis in the KKAy mice model and in CFs induced by TGF-β1. Compared with C57BL/6J mice, body weight and blood glucose were increased in KKAy mice. After 16 weeks of TSF treatment, body weight was markedly decreased and blood glucose was not statistically different from KKAy mice. Collagen deposition in the heart developed in KKAy mice and was inhibited after 16 weeks of treatment with TSF. *In vitro* experiments also revealed that TSF attenuated fibrosis damage.

Transforming growth factor-β1 is expressed in many tissues and varieties of cells, and its signaling pathway is involved in the expression of fibrogenic factors, including collagens I and III and α-SMA (18). Moreover, TGF-β1 is a crucial mediator of fibroblast activation and diseased heart fibrosis (3), and exerts its biologic effect through Smad signal transduction (19). In the presence of Smad3, TGF-β1 can induce the contraction of collagen lattice and the expression of α-SMA (20). The activation of TGF-β1/Smad3 pathways in cardiac fibrosis can result in myofibroblast proliferation and a marked upregulation the expressions of collagens I and III (21). In addition, the activation of TGF-β1/Smad3 signaling also leads to the degradation of Smad7. Smad7 is an inhibitory Smad, and the overexpression of Smad7 prevents fibrosis *in vitro* and *in vivo* (5). Our results were consistent with the aforementioned reports. In the KKAy mouse model with myocardial fibrosis, we found the overexpression of collagens I and III and α-SMA, as well as the activation of TGF-β1 and p-Smad2/3 and the reduction of Smad7. TSF is a drug that treats kidney disease, especially renal fibrosis. Previous studies have suggested that TSF alleviates renal fibrosis by regulating TGF-β1/Smad3 (5). TGF-β/Smad is a common signaling pathway in the injury of multiple organs. In this study, we found that TSF decreased the expressions of the target genes of fibrosis markers. Additionally, TSF decreased the expressions of TGF-β1 and p-Smad2/3, resulting in a significant reduction in both mRNA and protein levels. The expression of smad7 was increased with TSF treatment. Thus, it is conceivable that TSF alleviates myocardial fibrosis by inhibiting the TGF-β1/Smad signaling pathway. Fibroblasts are one of the major cytoeffectors of cardiac fibrosis. They are highly sensitive to TGF-βs, activate Smad-dependent signaling cascades, and are very much involved in regulating fibrosis transcriptional programs (18). In experiments with cultured CFs, TGF-β1 inhibits myocardial fibroblast apoptosis through Smad3 and extracellular signal-regulated kinase 1/2 (22). In our study, we established a TGF-β1-induced myocardial fibroblast fibrosis injury model, and the expression of the Smad downstream fibrosis gene was significantly increased compared with the control group, but after TSF treatment, the fibrosis indexes were significantly decreased, suggesting that TSF can also alleviate fibrosis damage *in vivo*. Taken together, TSF seems to attenuate myocardial fibrosis by regulating the TGF-β/Smad signaling pathway.

Both Wnt/β-catenin and TGF-β1 signaling have been found to stimulate and coordinate with each other, which plays an important role in the process of fibrosis (23). The canonical Wnt/β-catenin pathway is involved in fibroblast activation and proliferation (24) with Wnt1 being the actual promoter of fibrosis. Under normal conditions, the Wnt ligand is absent, and cytoplasmic β-catenin is phosphorylated by a destruction complex, including Axin adenomatous polyposis coli (APC) protein and glycogen synthase kinase (GSK)-3β, which is then ubiquitinated and destroyed by the proteasome. Therefore, β-catenin is kept at a low level. In contrast, when Wnt is present, its proteins transmit signals through the plasma membrane by interacting with the members of the frizzled (FZD) protein family and LRP5/6; the activation of the messy protein causes a separation of GSK-3β from the complex, which results in an inability to phosphorylate β-catenin. β-catenin is not degraded and translocated to the nucleus where it upregulates a wide range of target genes, such as MMP7 (25–28).

Matrix metalloproteinases (MMPs), a family of zinc-dependent endopeptidases, are involved in myocardial interstitial tissue changes in diseased atria (29). Myocardial fibrosis both in human patients and animal models has been found to be accompanied by increased MMP activity (30). Our *in vivo* experiments showed that Wnt1, active-β-catenin, and MMP7 protein expression were increased in KKAy mice. TSF inhibited changes in the expression of Wnt/β-catenin-related proteins that were observed in the myocardia of KKAy mice, indicating that the inhibition of Wnt/β-catenin-driven myocardial fibrosis may be another mechanism associated with the protective effect of TSF on myocardial fibrosis. Furthermore, it has been reported that the loss of the Wnt ligand reduces TGF-β expression and thus reducing the fibrotic response (31). Smad7, Axin, and E3 ubiquitin ligases form complexes after the activation of Wnt ligands, which facilitate Smad7 degradation. Smad7 also induces the ubiquitination and degradation of β-catenin by binding to Smurf 2 (32). In the TGF-β/Smad2/3 pathway, Smad7 blocks the expression of Smad3 and plays a negative feedback regulatory role (33). In our study, TSF promoted the expression of Smad7, thereby inhibiting the TGF-β/Smad2/3 pathway and promoting the phosphorylation of β-catenin. TSF is a Chinese medicine compound and contains monomers, such as calycosin, quercetin, and kaempferol, which have been found to act on the fibrosis pathway. Kaempferol is found in the TSF herbs astragalus root and burning bush twig. It has been shown to inhibit fibroblast collagen synthesis, proliferation, and activation, and inhibit TGF-β/Smads signaling by selectively binding TGF-β RI and significantly downregulating Smad2 and Smad3 phosphorylation in a dose-dependent manner (34). Quercetin has been reported to have protective effects on the heart because of its antioxidant, anti-inflammatory, and other biological properties (35). Albadrani et al. revealed that quercetin upregulates Smad7 and has an anti-fibrosis effect (36). Quercetin is contained in TSF herbs, such as rhubarb root and rhizome, notoginseng root, cornus fruit, burning bush twig, and astragalus root, so the TSF regulation of Smad7 is dependent on quercetin. Astragalus root contains calycosin, which can also upregulate Smad7, thereby inhibiting fibrosis (37). Therefore, perhaps the two monomers in TSF are involved in regulating Smad7. In conclusion, we speculate that Smad7 may have mediated the interaction between TGF-β/Smad2/3 and Wnt/β-catenin in the TSF treatment of myocardial fibrosis (Figure 6).

The results of this study indicate that TSF appears to be effective in attenuating myocardial fibrosis both *in vitro* and *in vivo*. Thus, TSF may be a potential clinical treatment for patients with diabetic cardiomyopathy. However, the limitation of our current research is that although we have elucidated the pathway, we have yet to clarify the target. Therefore, one of our future studies will be to demonstrate the specific target of TSF in alleviating myocardial fibrosis.

## Conclusion

The present study revealed that TSF may alleviate myocardial fibrosis in KKAy mouse models by inhibiting TGF-β/Smad and Wnt/β-catenin signaling pathways. These findings may contribute to our understanding of the protective effect of TSF on myocardial fibrosis and further enrich our understanding of the mechanisms of myocardial fibrosis in diabetes.

## Data Availability Statement

The original contributions presented in the study are included in the article/Supplementary Material, further inquiries can be directed to the corresponding author/s.

## Ethics Statement

The animal study was reviewed and approved by Beijing National Proteome Science Center Animal Management Ethics Committee.

## Author Contributions

PL, TZ, and LH conceived and designed the experiments. LH and YuzW performed the experiments. LH and YuyW analyzed the data. LM contributed materials and analysis tools. LH and TZ wrote the paper. All authors contributed to the article and approved the submitted version.

## Funding

This study was supported by the National Natural Science Foundation of China [Grant Nos: 81973627, 82074221, and 81620108031] and Beijing Natural Science Foundation [Grant No: 7212195].

## Conflict of Interest

The authors declare that the research was conducted in the absence of any commercial or financial relationships that could be construed as a potential conflict of interest.

## Publisher's Note

All claims expressed in this article are solely those of the authors and do not necessarily represent those of their affiliated organizations, or those of the publisher, the editors and the reviewers. Any product that may be evaluated in this article, or claim that may be made by its manufacturer, is not guaranteed or endorsed by the publisher.
