# Supplementary figures and images for: Tangshen Formula Improves Diabetes-Associated Myocardial Fibrosis by Inhibiting TGF-β/Smads and Wnt/β-Catenin Pathways
Source: Front Med (Lausanne). 2021 Dec 6;8:732042. doi: 10.3389/fmed.2021.732042 (PMC8687440; doi:10.3389/fmed.2021.732042)

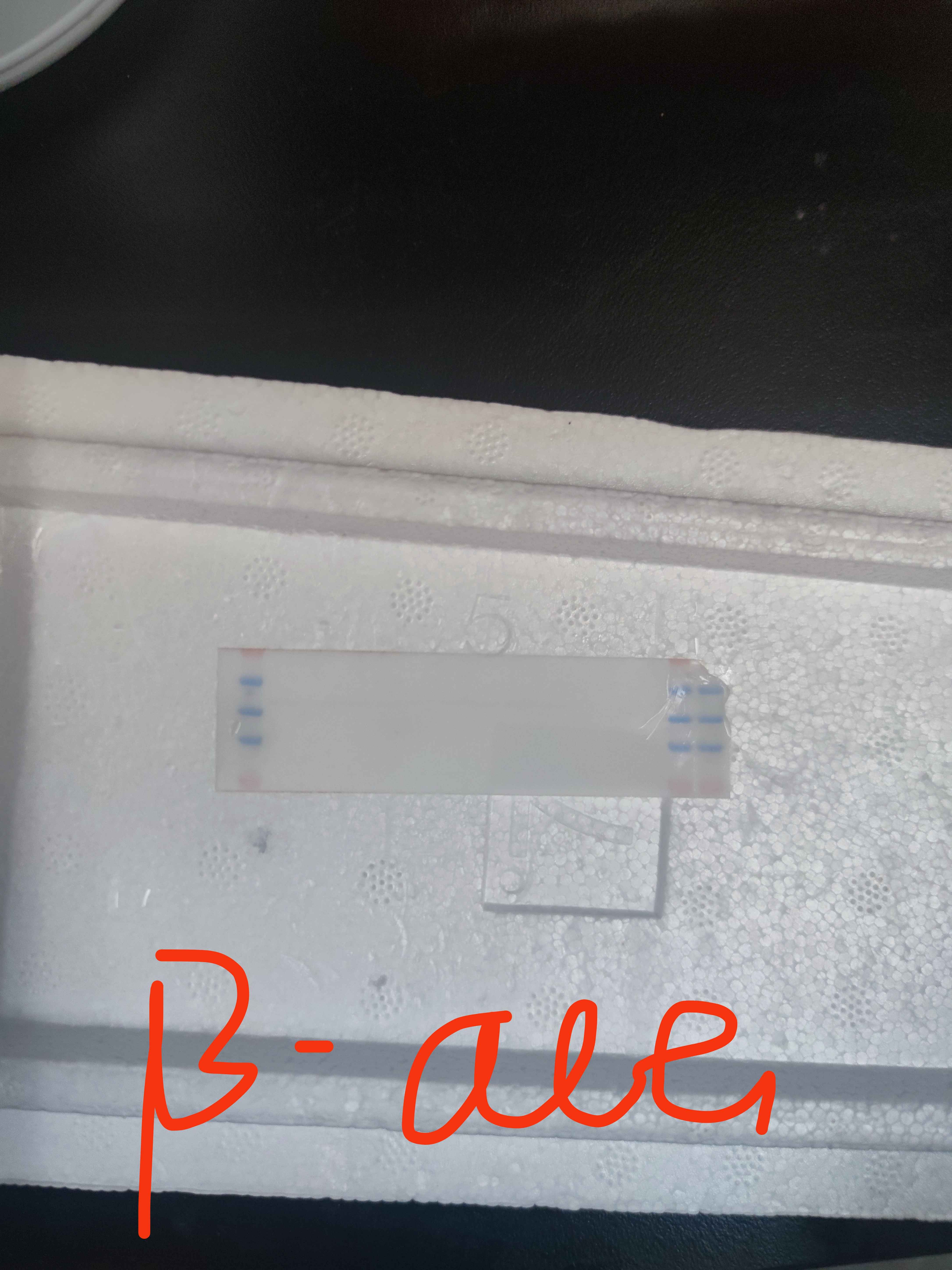

Supplement: Supplementary file 2 [file Data_Sheet_2.ZIP › wb/Figure 2 bactin 1.jpg]

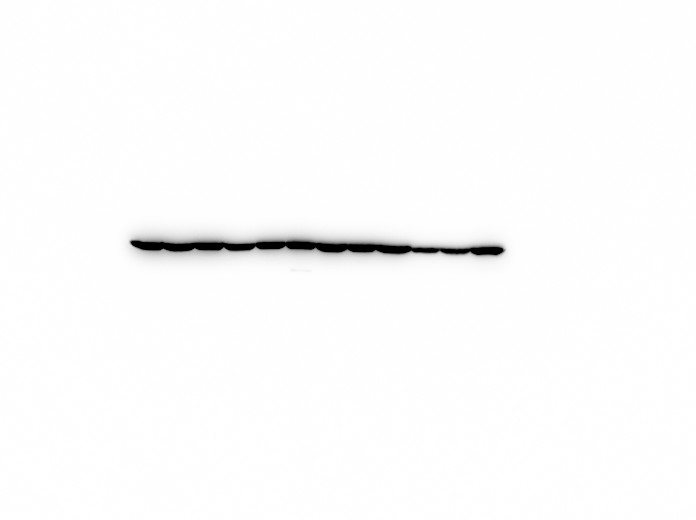

Supplement: Supplementary file 2 [file Data_Sheet_2.ZIP › wb/Figure 2 bactin2.jpg]

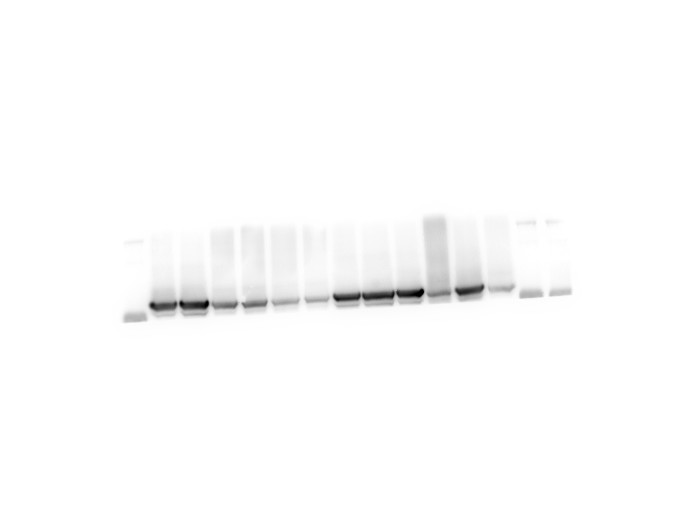

Supplement: Supplementary file 2 [file Data_Sheet_2.ZIP › wb/Figure 2 col3 1.jpg]

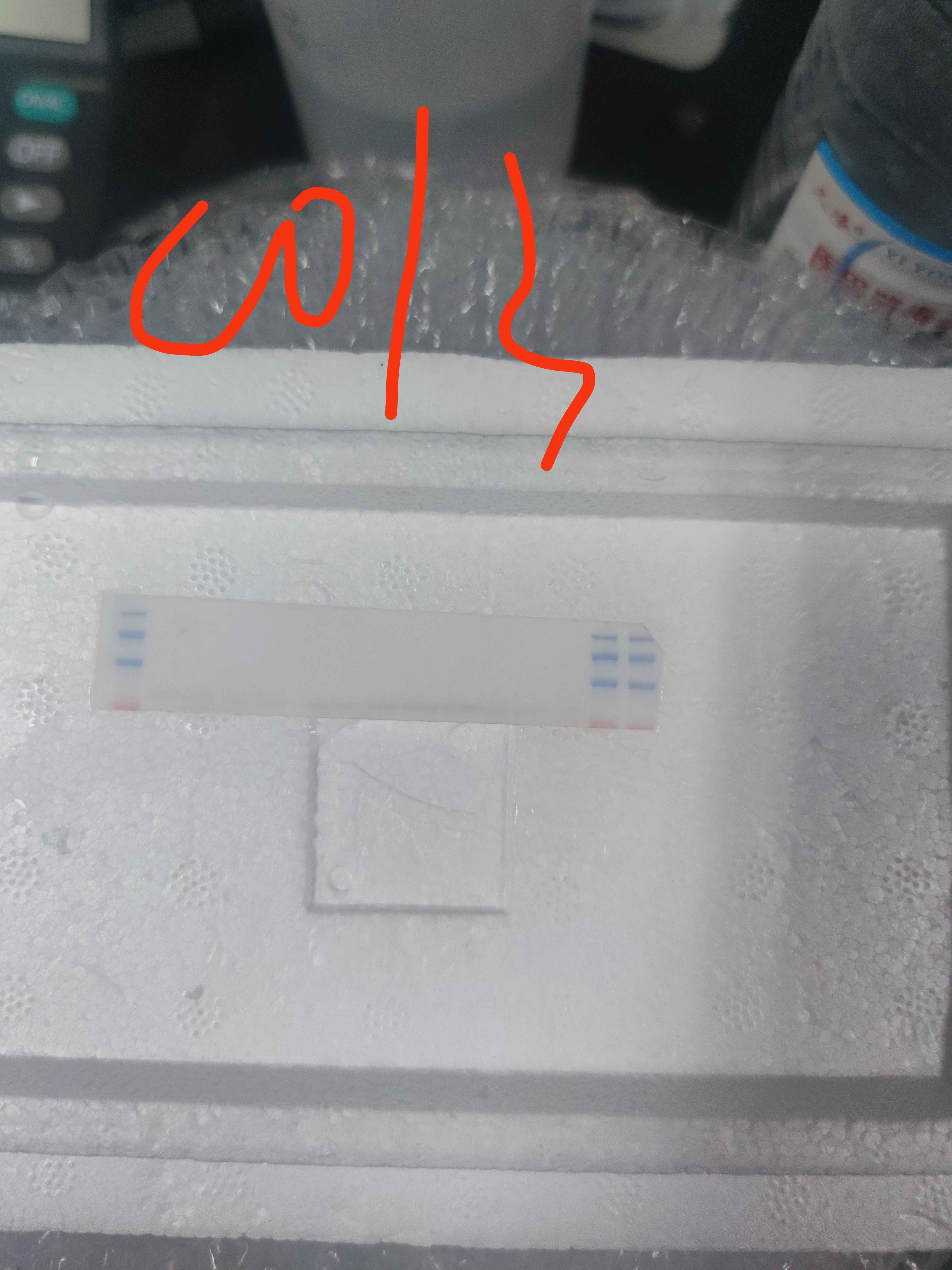

Supplement: Supplementary file 2 [file Data_Sheet_2.ZIP › wb/Figure 2 col3 2.jpg]

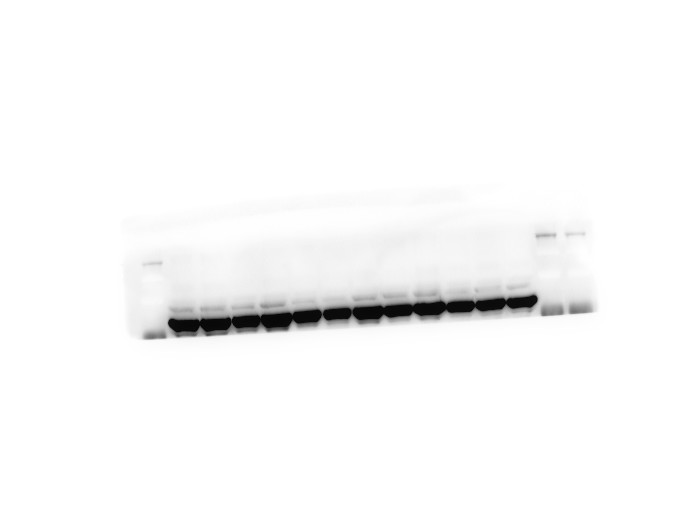

Supplement: Supplementary file 2 [file Data_Sheet_2.ZIP › wb/Figure 2 col3 3.jpg]

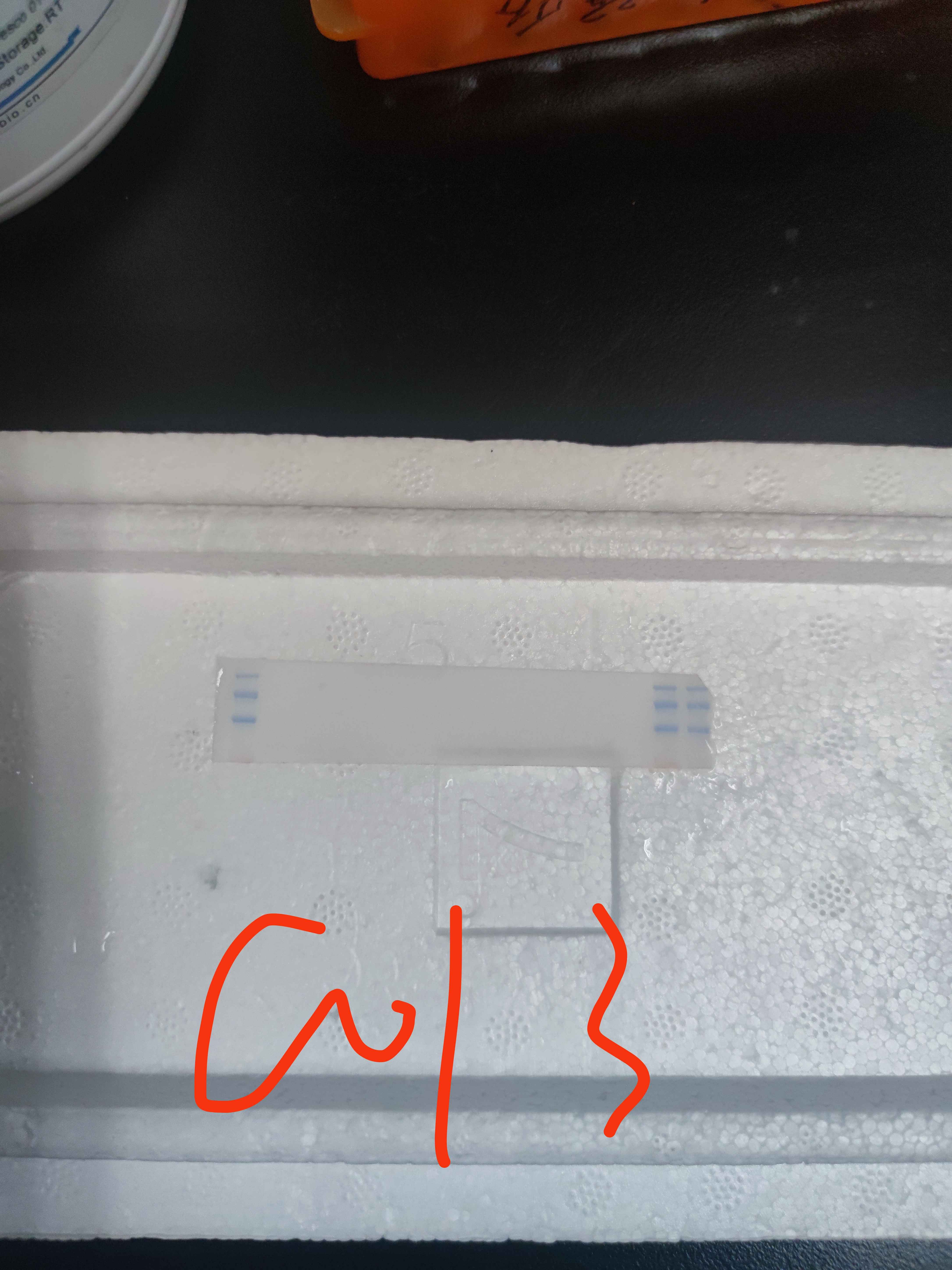

Supplement: Supplementary file 2 [file Data_Sheet_2.ZIP › wb/Figure 2 col3 4.jpg]

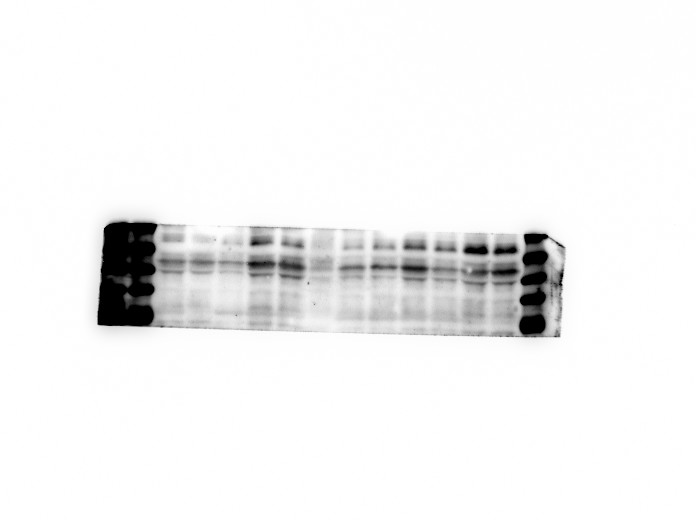

Supplement: Supplementary file 2 [file Data_Sheet_2.ZIP › wb/Figure2 a-sma 1.jpg]

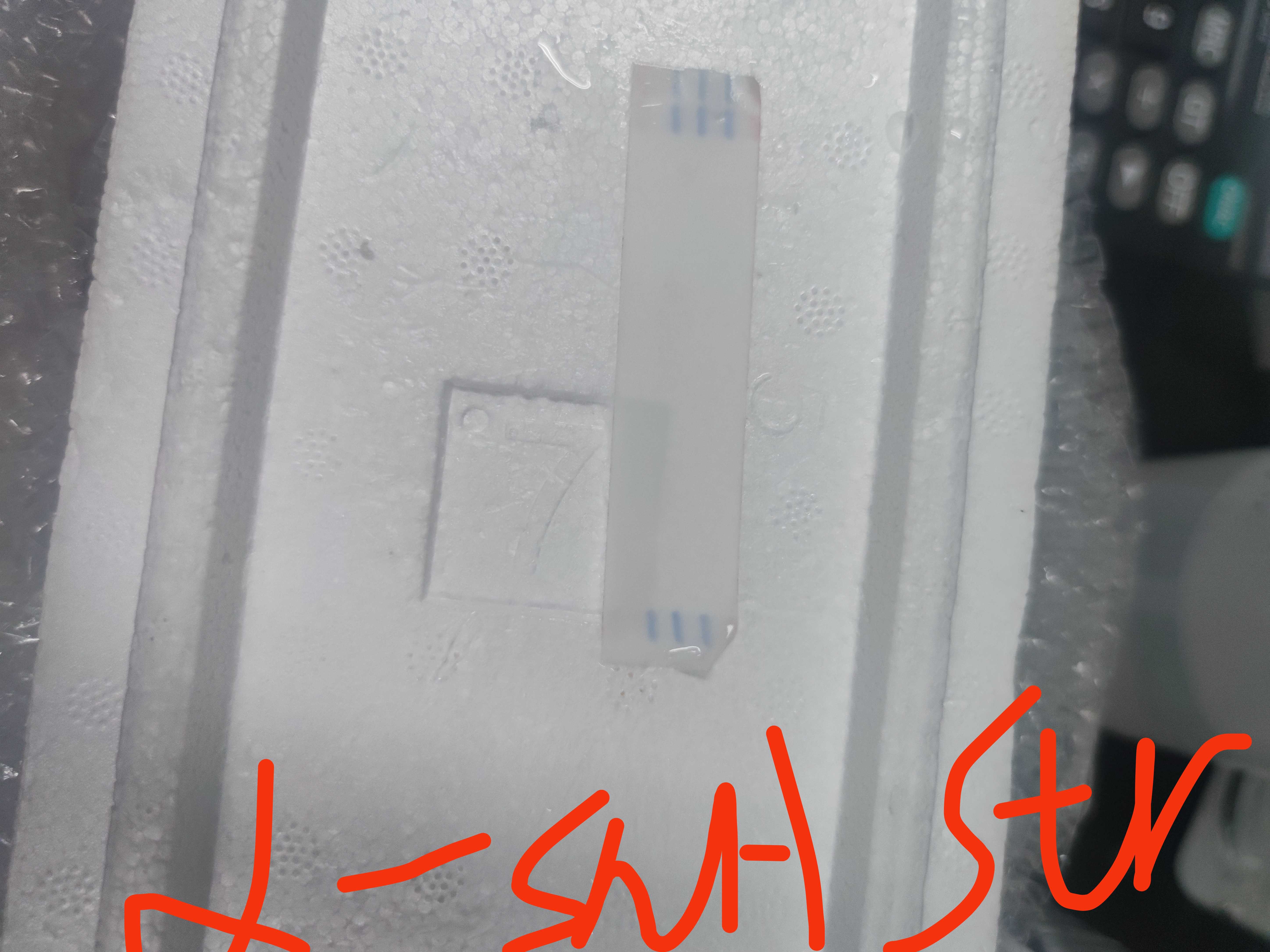

Supplement: Supplementary file 2 [file Data_Sheet_2.ZIP › wb/Figure2 a-sma 2.jpg]

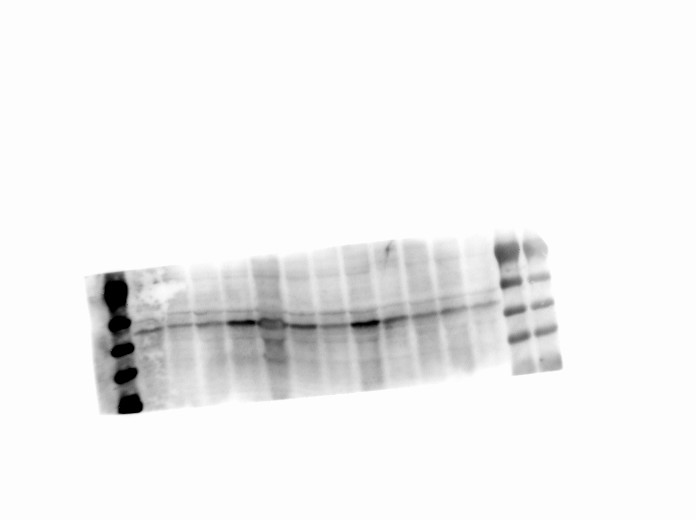

Supplement: Supplementary file 2 [file Data_Sheet_2.ZIP › wb/Figure2 a-sma 3.jpg]

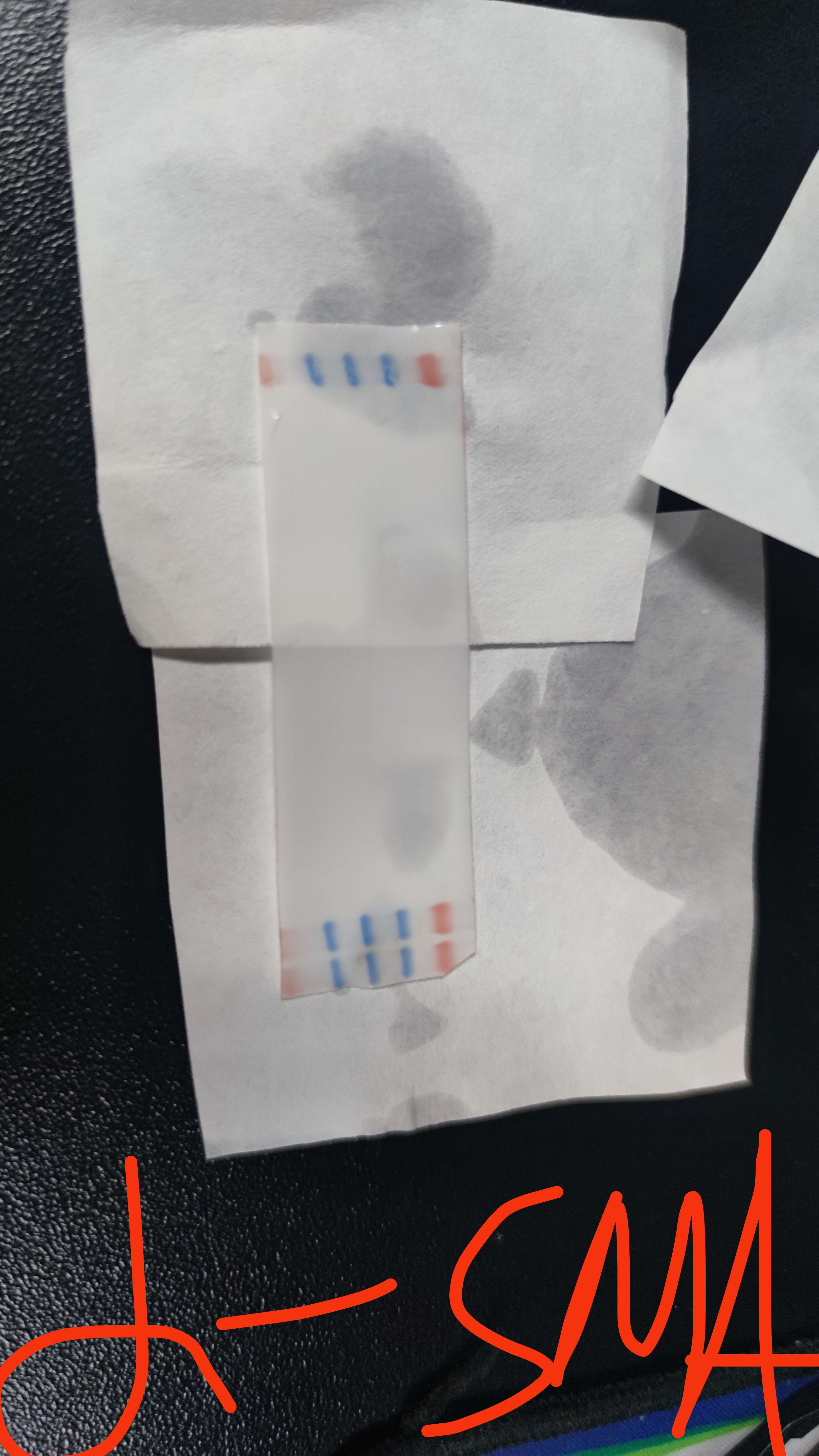

Supplement: Supplementary file 2 [file Data_Sheet_2.ZIP › wb/Figure2 a-sma 4.jpg]

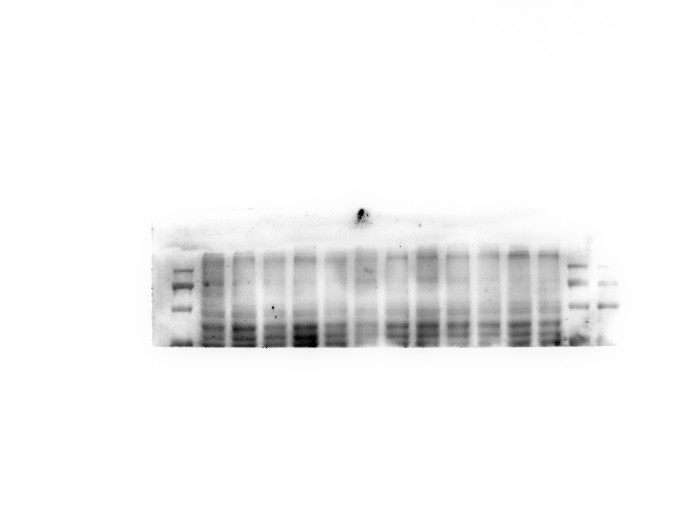

Supplement: Supplementary file 2 [file Data_Sheet_2.ZIP › wb/Figure2 col1 1.jpg]

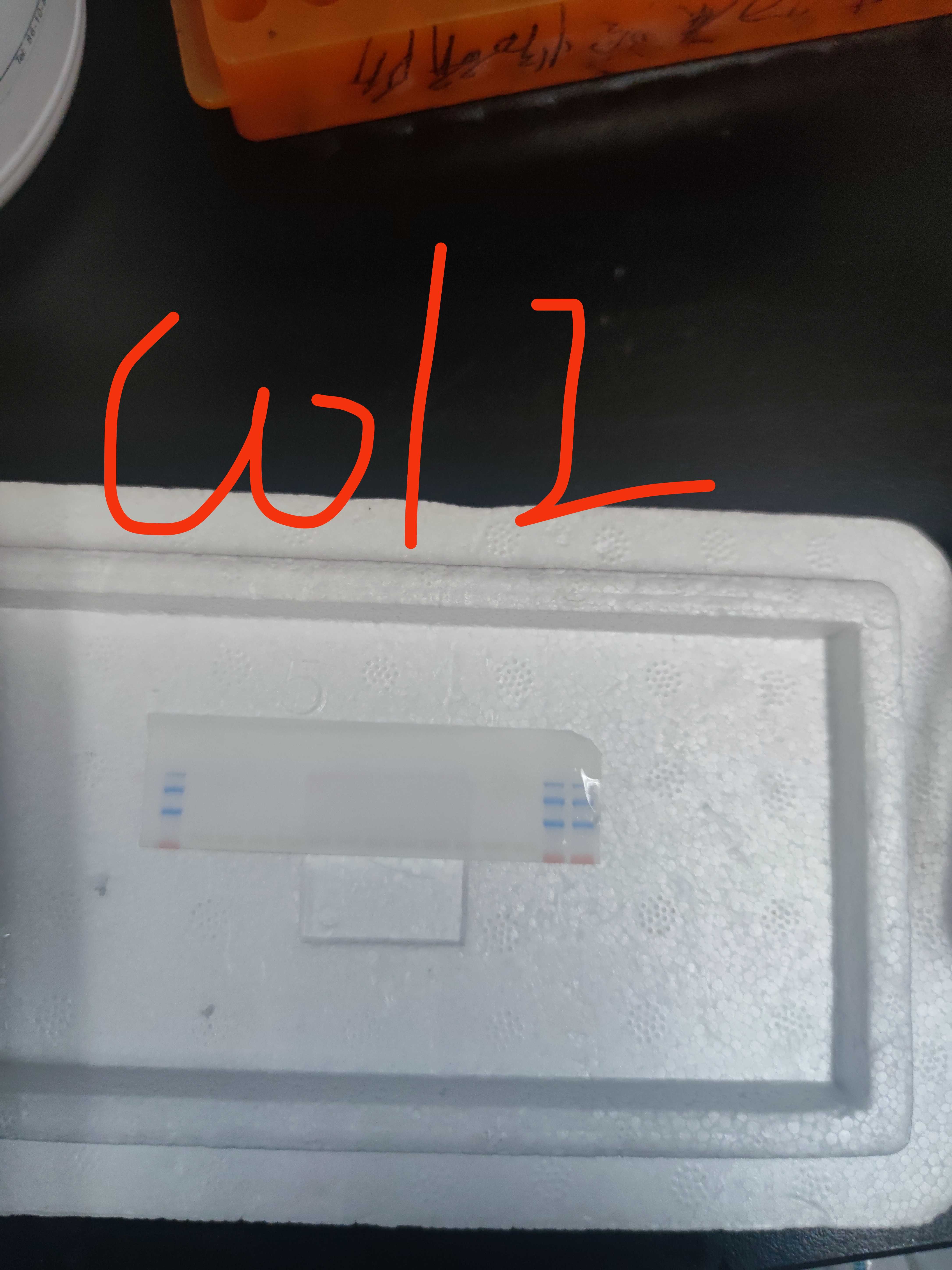

Supplement: Supplementary file 2 [file Data_Sheet_2.ZIP › wb/Figure2 col1 2.jpg]

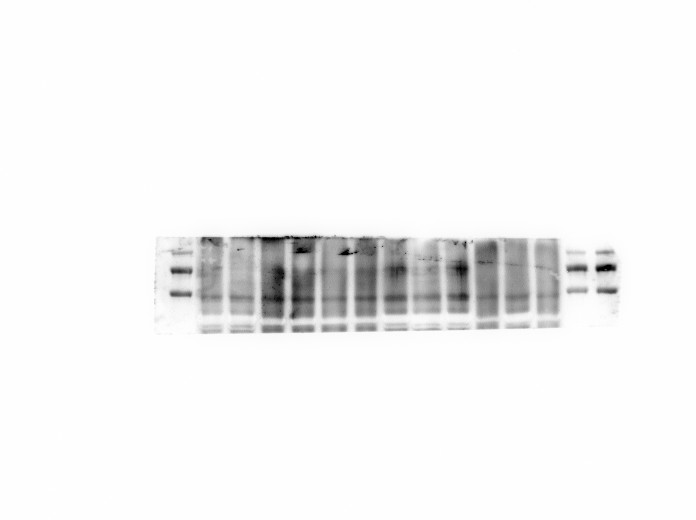

Supplement: Supplementary file 2 [file Data_Sheet_2.ZIP › wb/Figure2 col1 3.jpg]

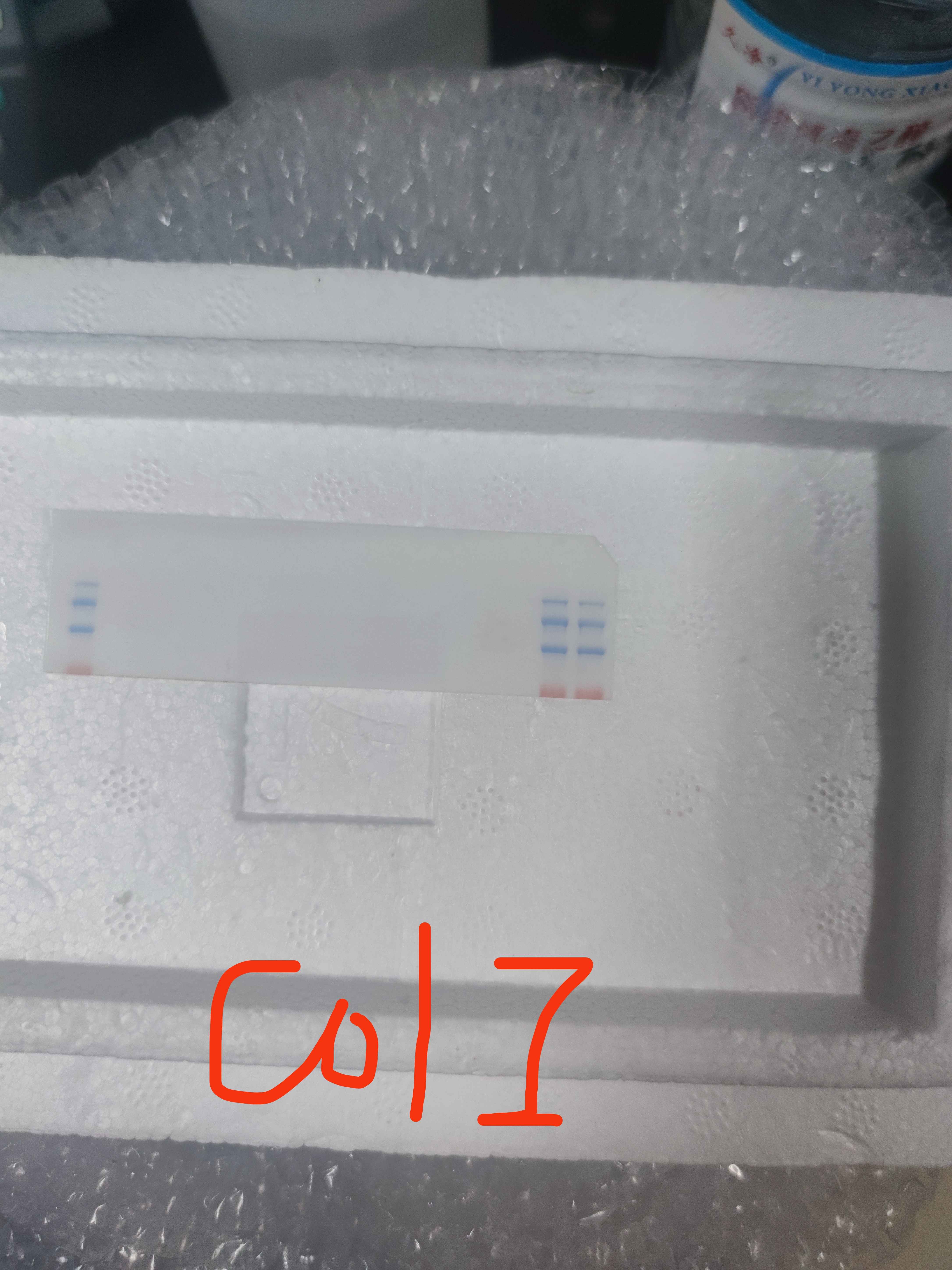

Supplement: Supplementary file 2 [file Data_Sheet_2.ZIP › wb/Figure2 col1 4.jpg]

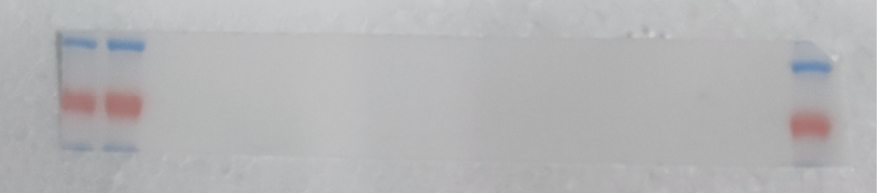

Supplement: Supplementary file 2 [file Data_Sheet_2.ZIP › wb/figure 3-TGF-a┬1(1).png]

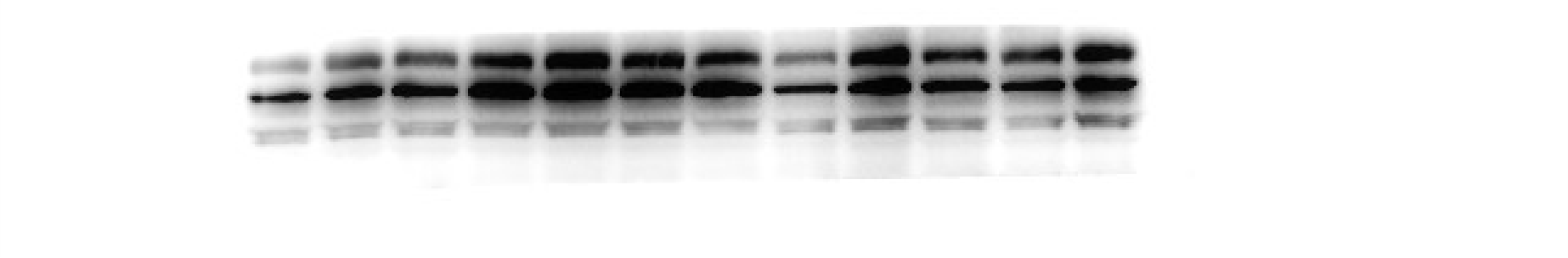

Supplement: Supplementary file 2 [file Data_Sheet_2.ZIP › wb/figure 3-TGF-a┬1(2).png]

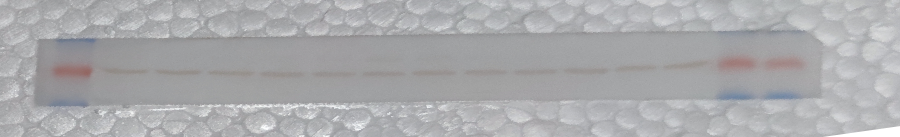

Supplement: Supplementary file 2 [file Data_Sheet_2.ZIP › wb/figure 3-TGF-a┬1(3).png]

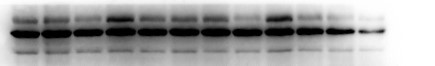

Supplement: Supplementary file 2 [file Data_Sheet_2.ZIP › wb/figure 3-TGF-a┬1(4).jpg]

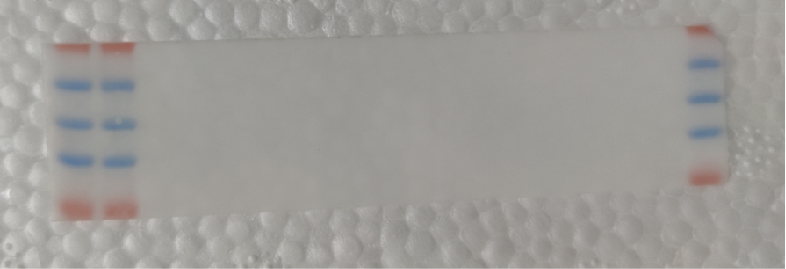

Supplement: Supplementary file 2 [file Data_Sheet_2.ZIP › wb/figure 3-Wnt1(1).png]

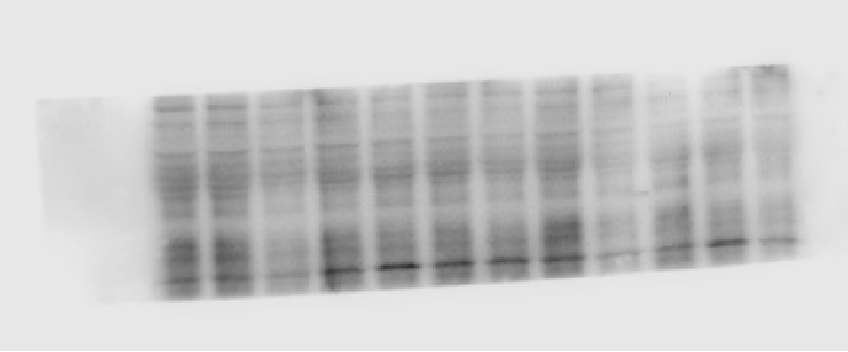

Supplement: Supplementary file 2 [file Data_Sheet_2.ZIP › wb/figure 3-Wnt1(2).png]

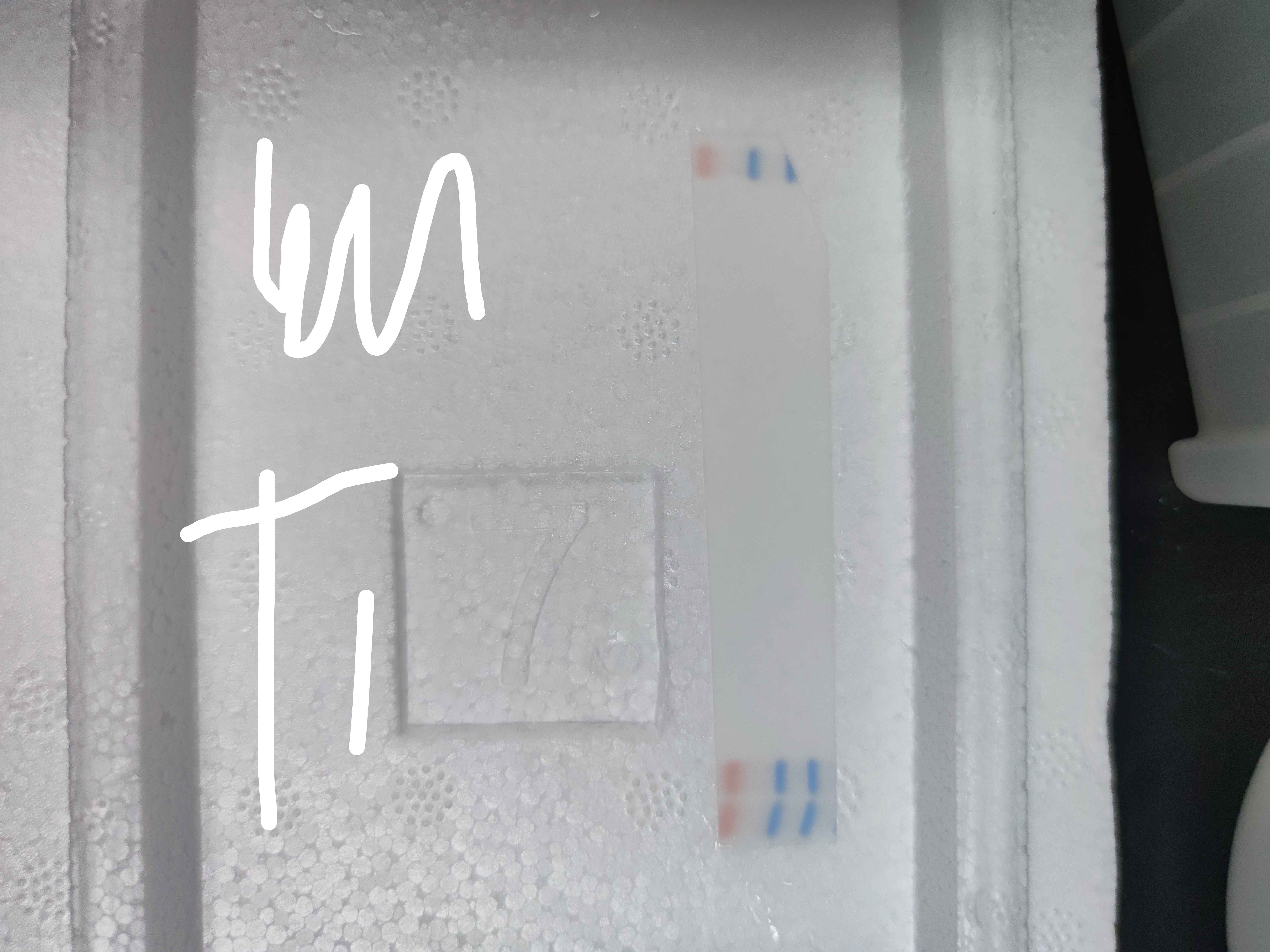

Supplement: Supplementary file 2 [file Data_Sheet_2.ZIP › wb/figure 3-Wnt1(3).jpg]

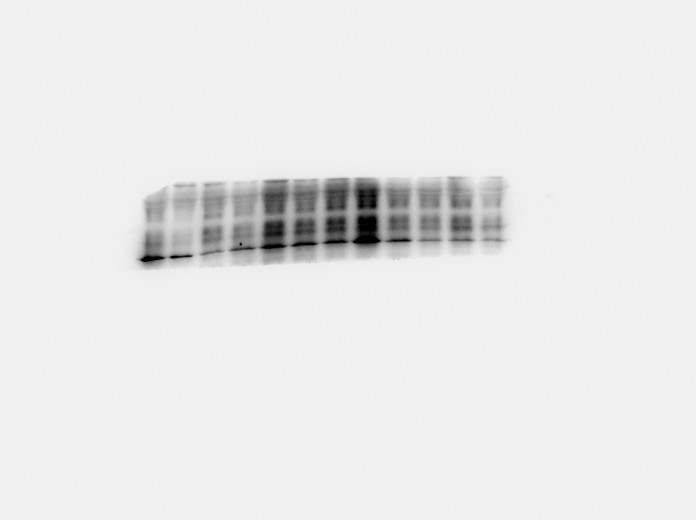

Supplement: Supplementary file 2 [file Data_Sheet_2.ZIP › wb/figure 3-Wnt1(4).jpg]

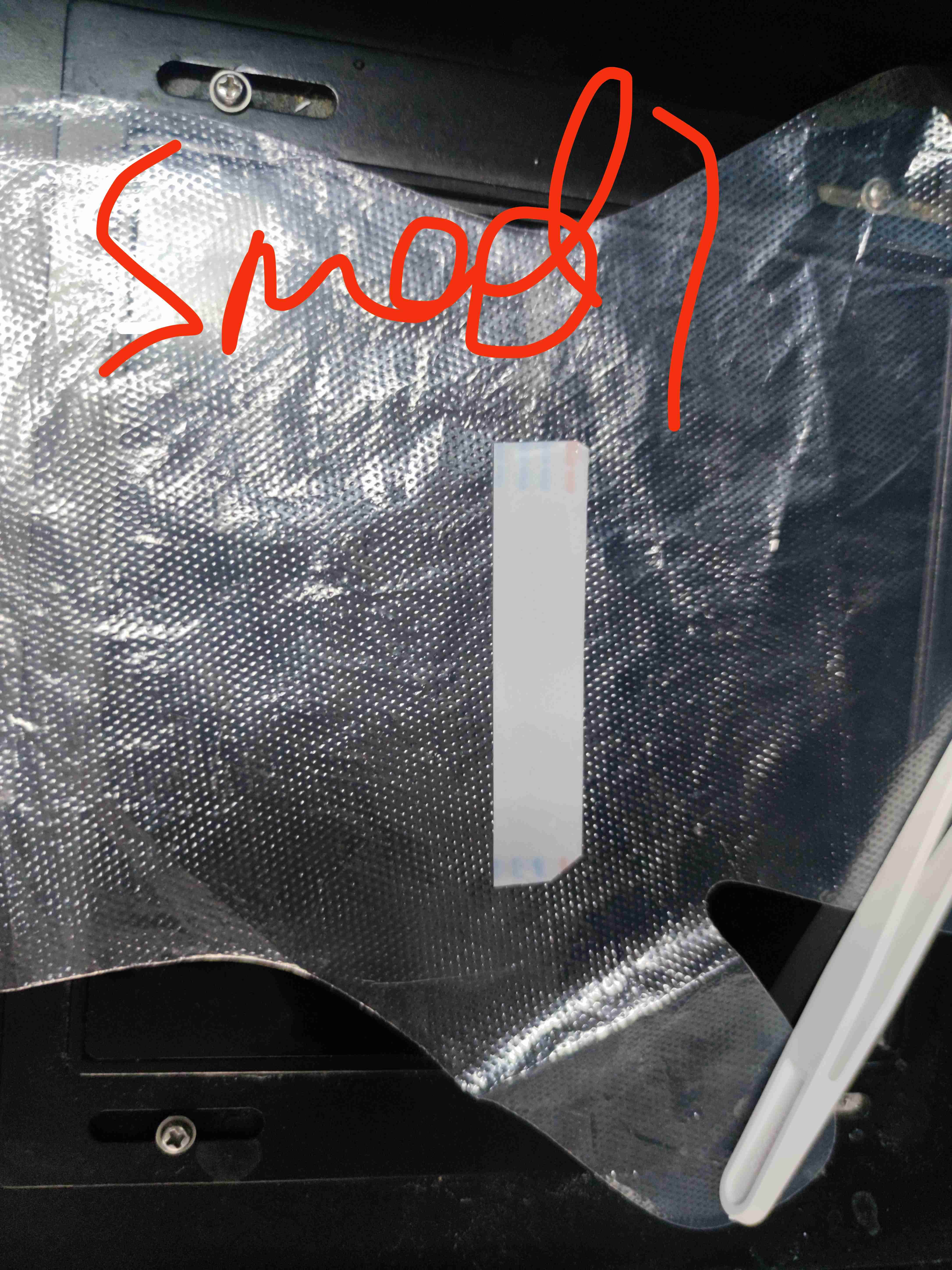

Supplement: Supplementary file 2 [file Data_Sheet_2.ZIP › wb/figure 3-smad7(1).jpg]

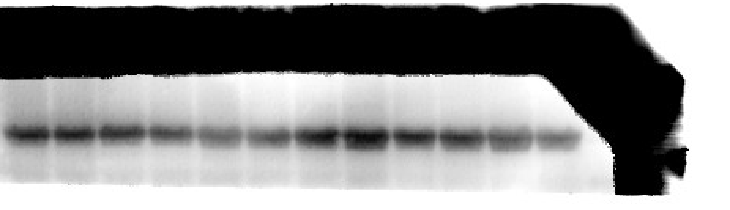

Supplement: Supplementary file 2 [file Data_Sheet_2.ZIP › wb/figure 3-smad7(1).png]

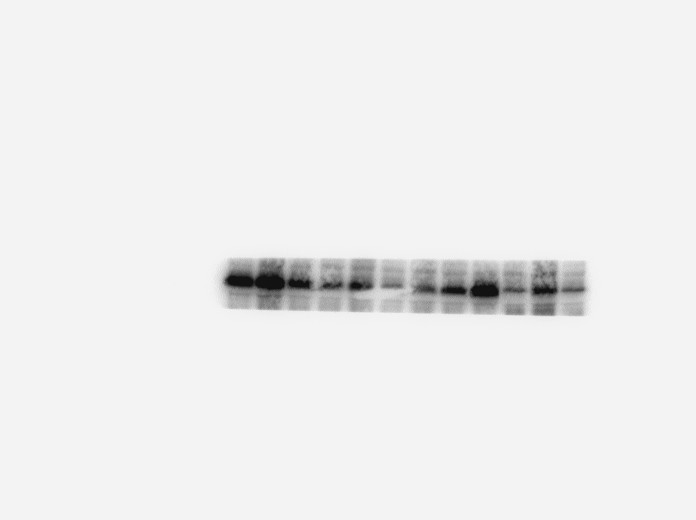

Supplement: Supplementary file 2 [file Data_Sheet_2.ZIP › wb/figure 3-smad7(3).jpg]

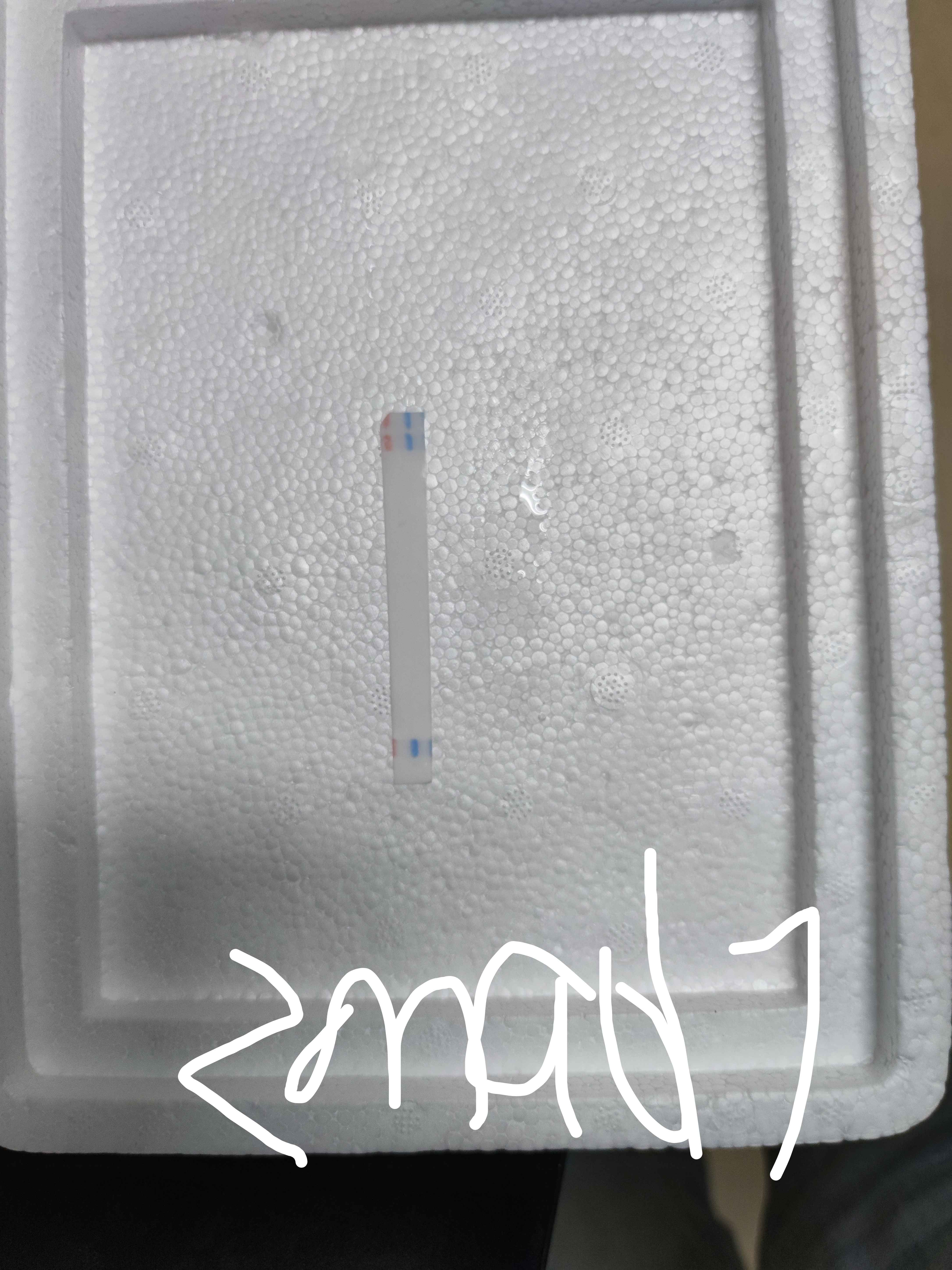

Supplement: Supplementary file 2 [file Data_Sheet_2.ZIP › wb/figure 3-smad7(4).jpg]

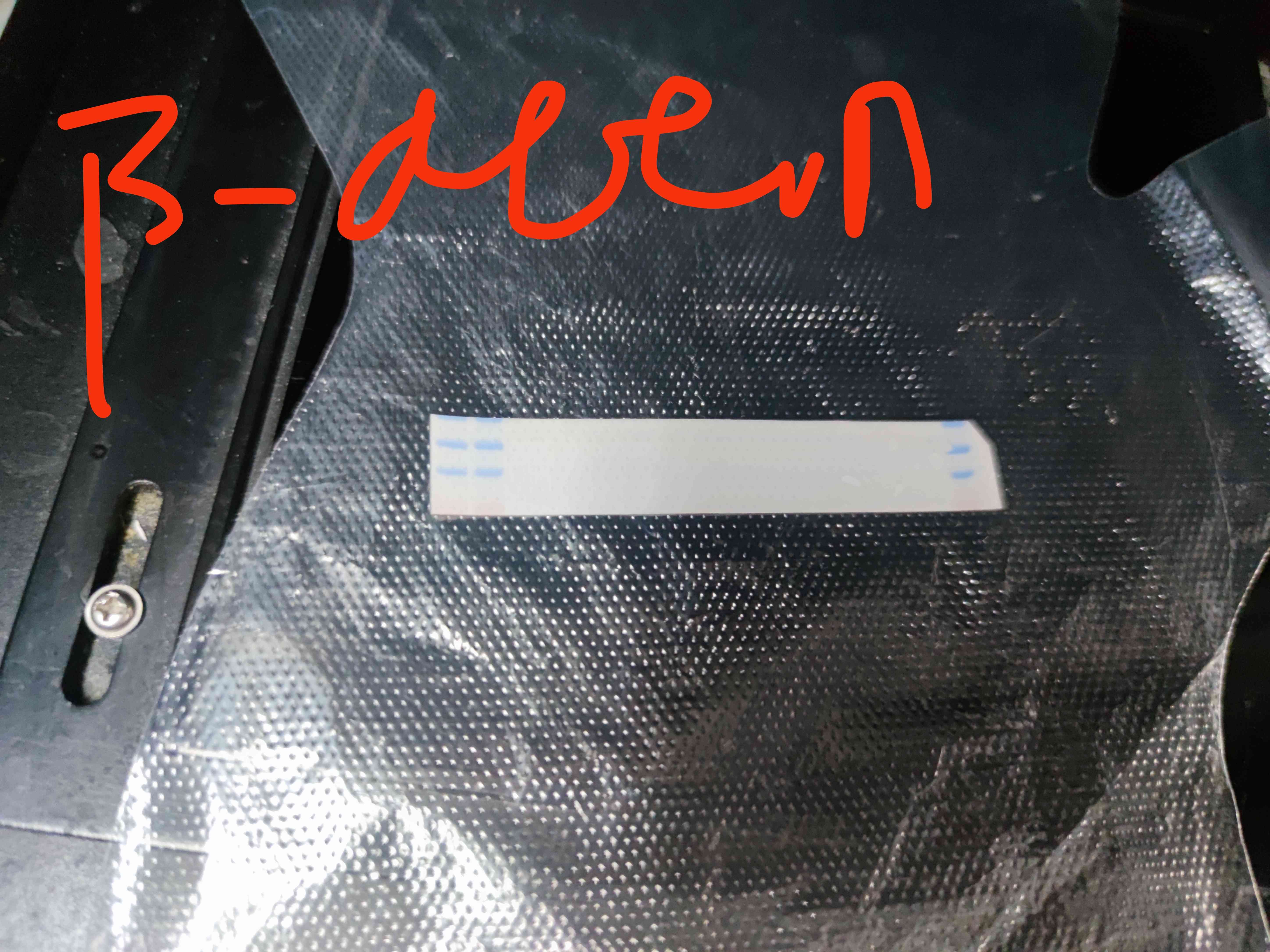

Supplement: Supplementary file 2 [file Data_Sheet_2.ZIP › wb/figure 3-a┬-actin(1).jpg]

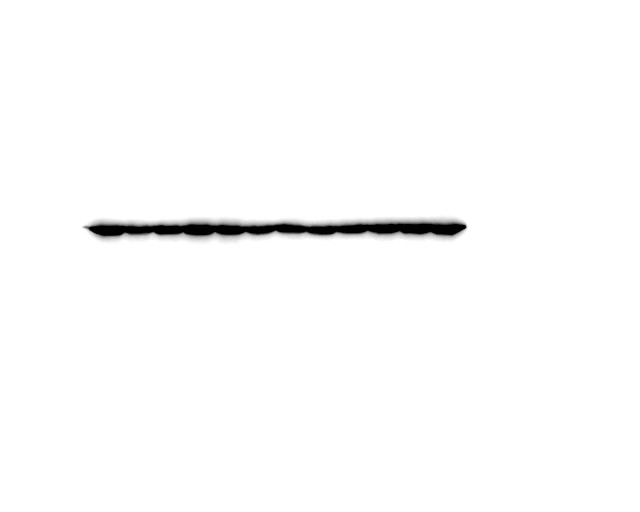

Supplement: Supplementary file 2 [file Data_Sheet_2.ZIP › wb/figure 3-a┬-actin(2).jpg]

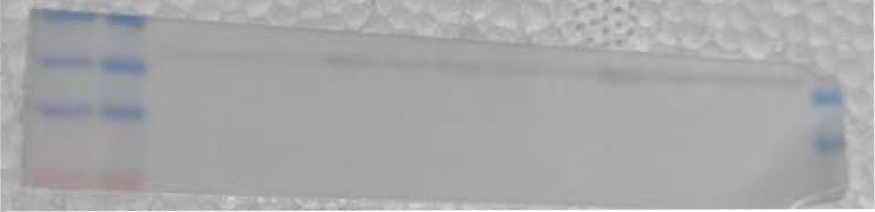

Supplement: Supplementary file 2 [file Data_Sheet_2.ZIP › wb/figure 3-a┬-actin(3).png]

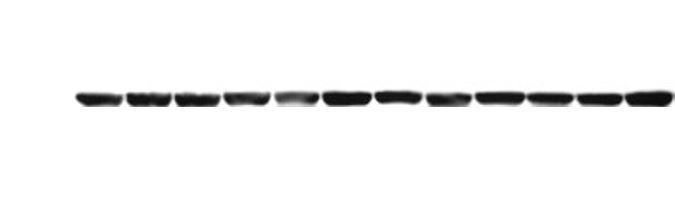

Supplement: Supplementary file 2 [file Data_Sheet_2.ZIP › wb/figure 3-a┬-actin(4ú⌐.JPG]

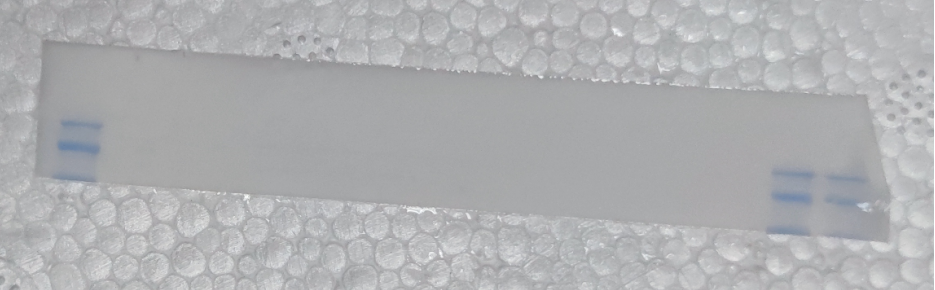

Supplement: Supplementary file 2 [file Data_Sheet_2.ZIP › wb/figure 4-FN(1).png]

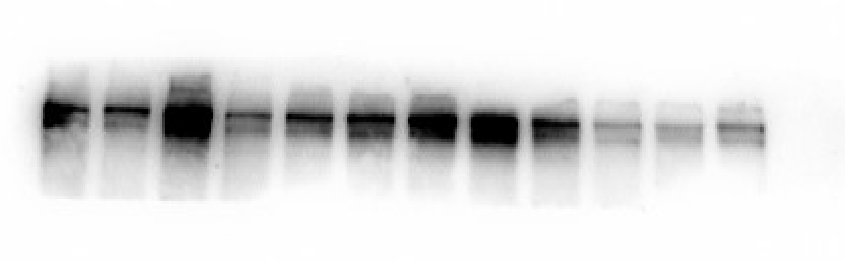

Supplement: Supplementary file 2 [file Data_Sheet_2.ZIP › wb/figure 4-FN(2).png]

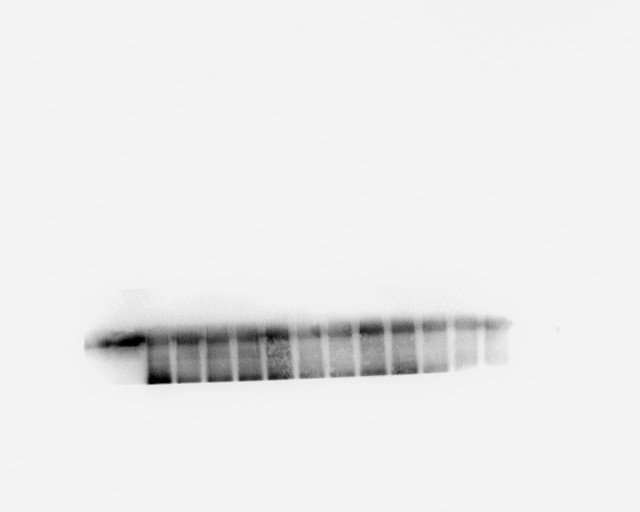

Supplement: Supplementary file 2 [file Data_Sheet_2.ZIP › wb/figure 4-FN(3).jpg]

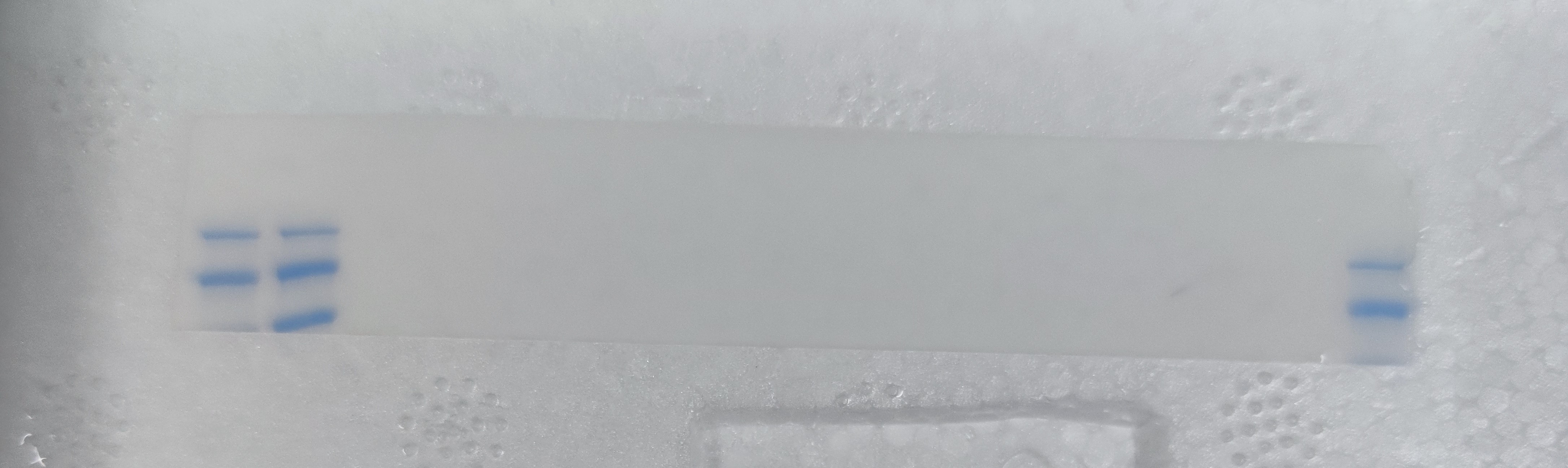

Supplement: Supplementary file 2 [file Data_Sheet_2.ZIP › wb/figure 4-FN(4).jpg]

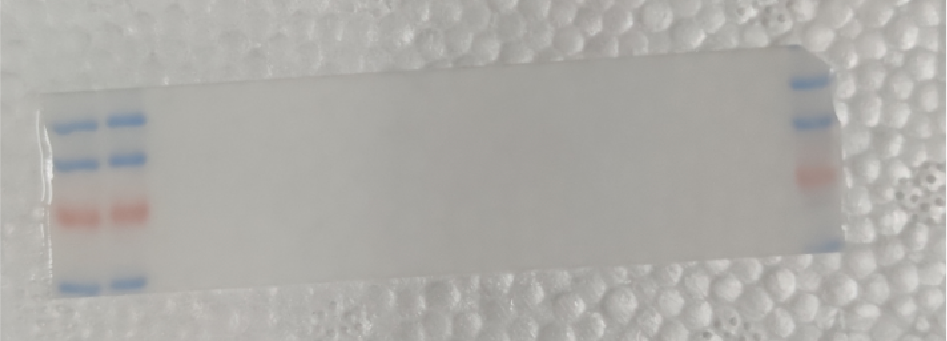

Supplement: Supplementary file 2 [file Data_Sheet_2.ZIP › wb/figure 4-MMP7(1).png]

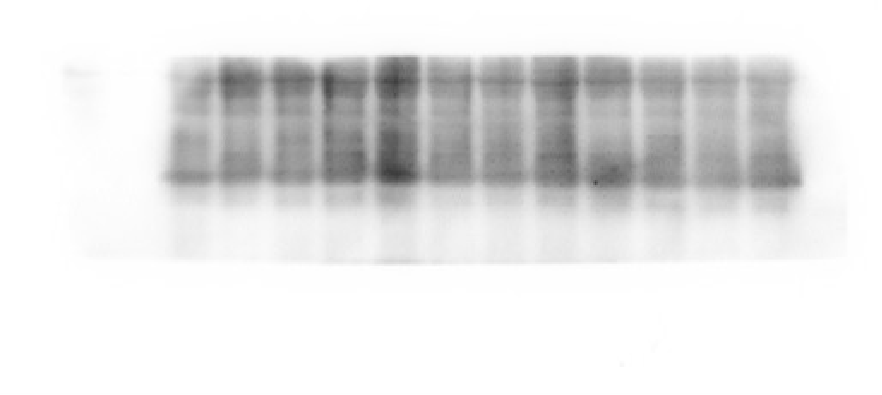

Supplement: Supplementary file 2 [file Data_Sheet_2.ZIP › wb/figure 4-MMP7(2).png]

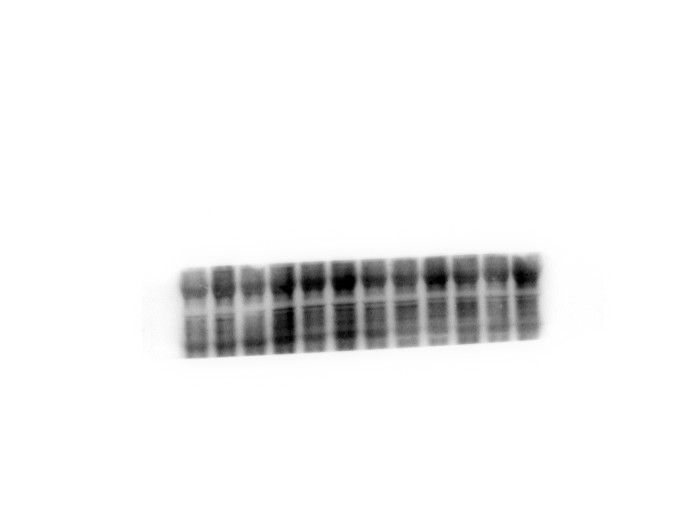

Supplement: Supplementary file 2 [file Data_Sheet_2.ZIP › wb/figure 4-MMP7(3).jpg]

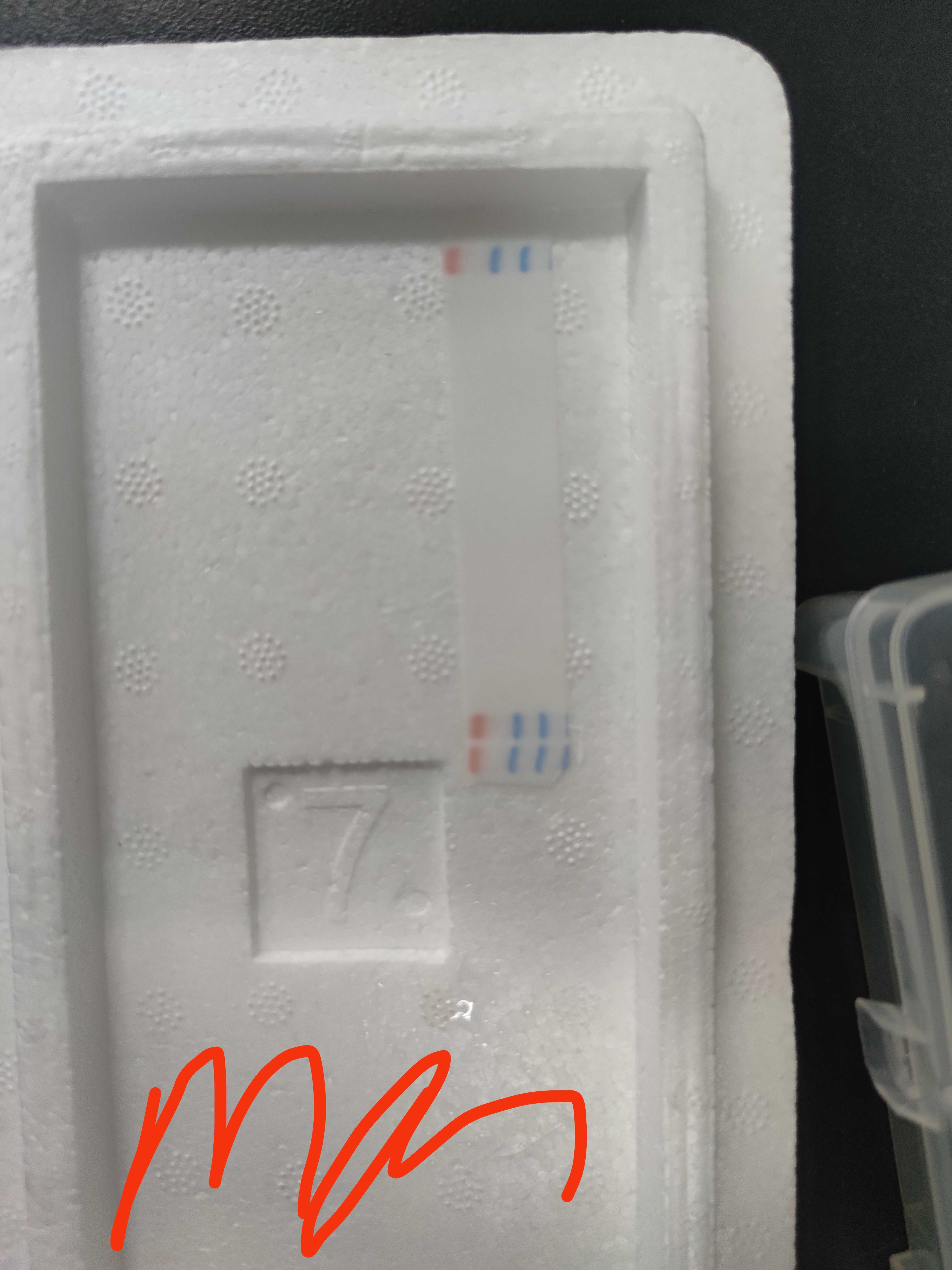

Supplement: Supplementary file 2 [file Data_Sheet_2.ZIP › wb/figure 4-MMP7(4).jpg]

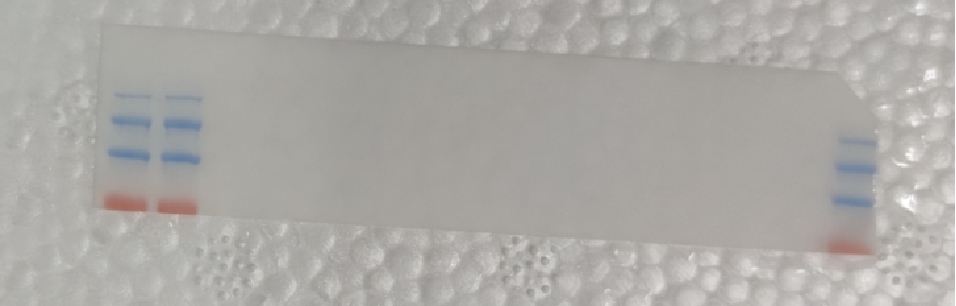

Supplement: Supplementary file 2 [file Data_Sheet_2.ZIP › wb/figure 4-active-a┬-catenin ú¿1ú⌐.png]

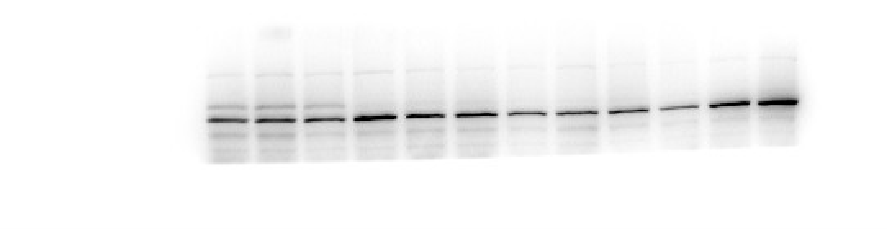

Supplement: Supplementary file 2 [file Data_Sheet_2.ZIP › wb/figure 4-active-a┬-catenin ú¿2ú⌐.png]

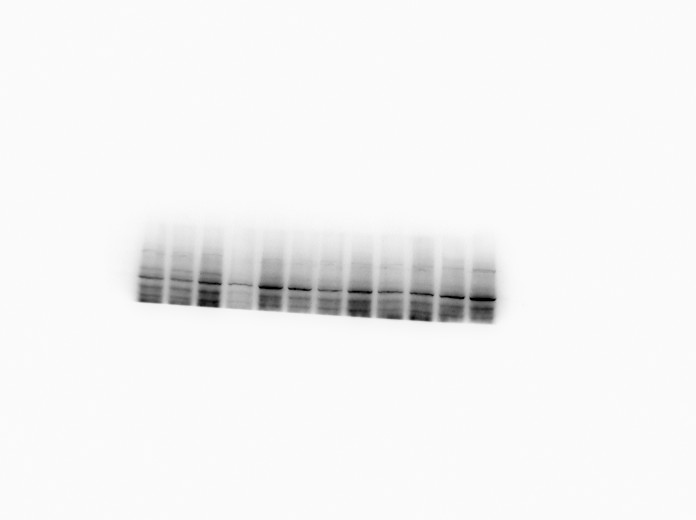

Supplement: Supplementary file 2 [file Data_Sheet_2.ZIP › wb/figure 4-active-a┬-catenin ú¿3ú⌐.jpg]

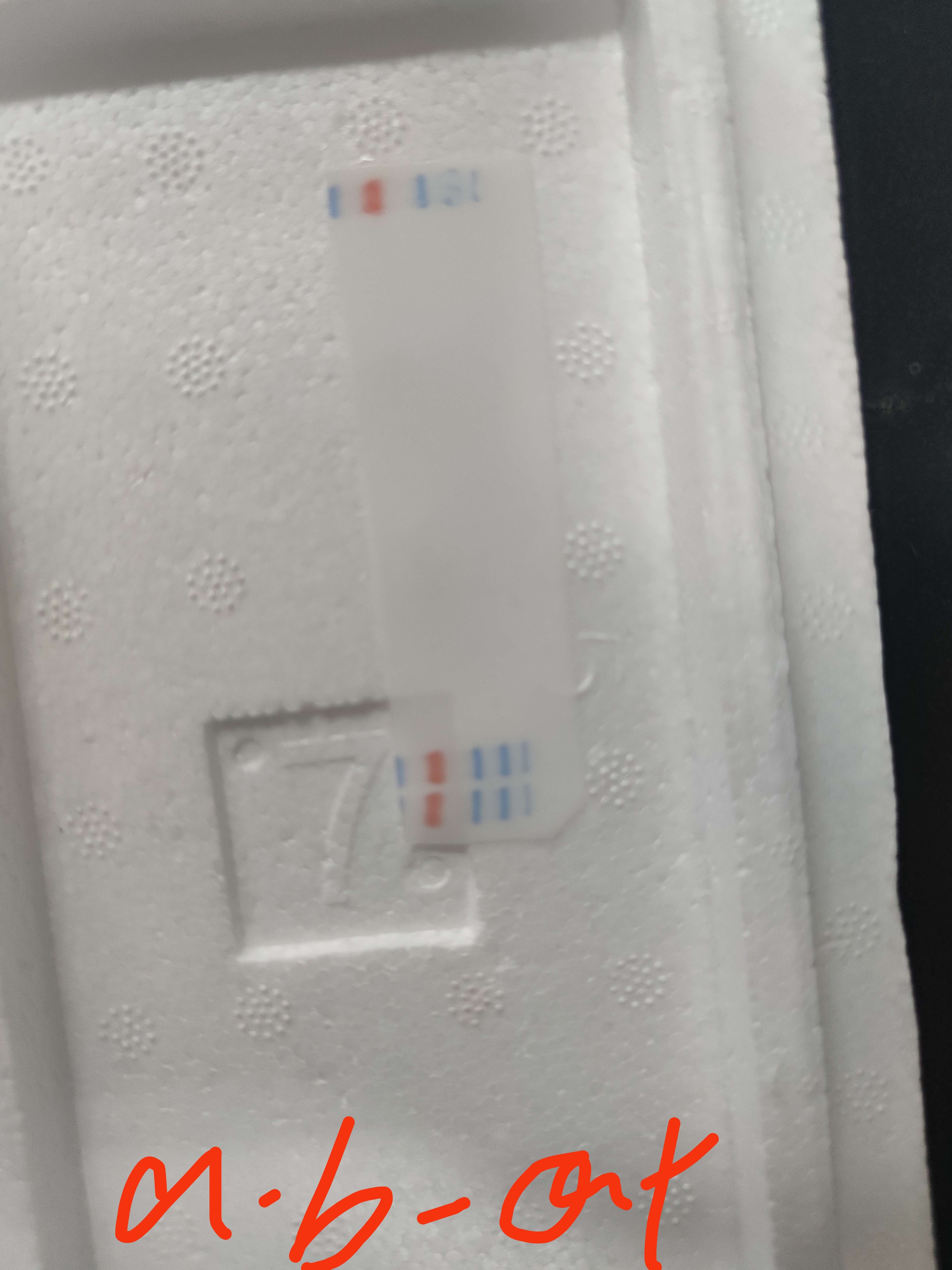

Supplement: Supplementary file 2 [file Data_Sheet_2.ZIP › wb/figure 4-active-a┬-catenin ú¿4ú⌐.jpg]

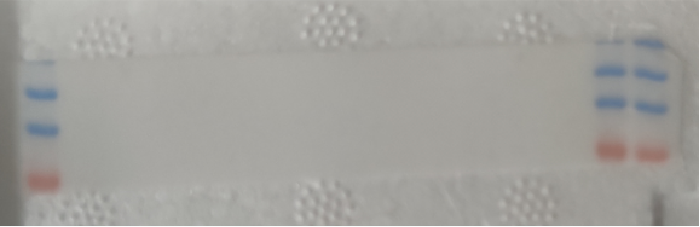

Supplement: Supplementary file 2 [file Data_Sheet_2.ZIP › wb/figure 4-a┬-actin(1).png]

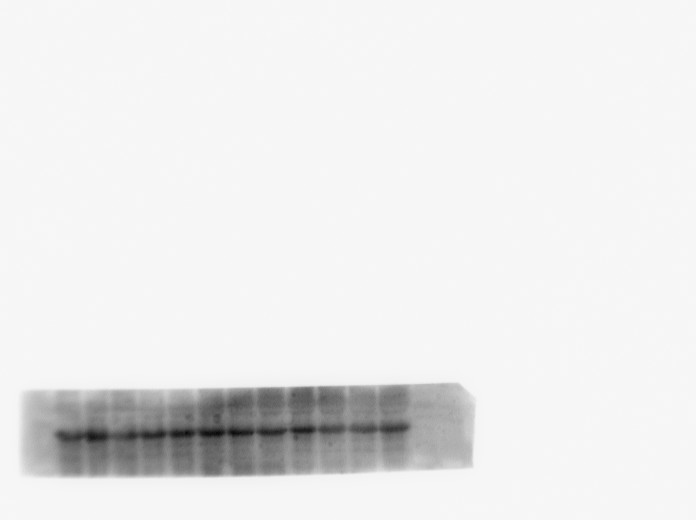

Supplement: Supplementary file 2 [file Data_Sheet_2.ZIP › wb/figure 4-a┬-actin(2).jpg]

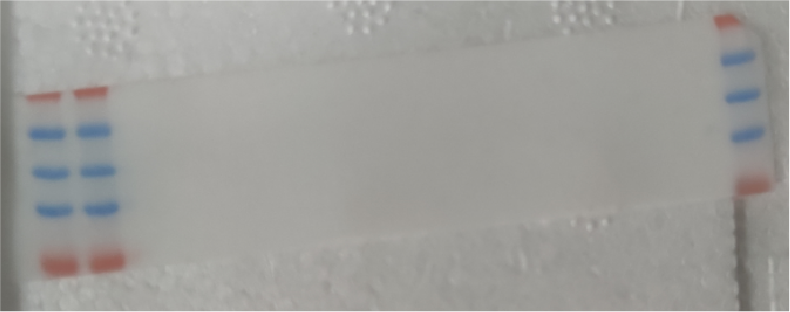

Supplement: Supplementary file 2 [file Data_Sheet_2.ZIP › wb/figure 4-a┬-actin(3).png]

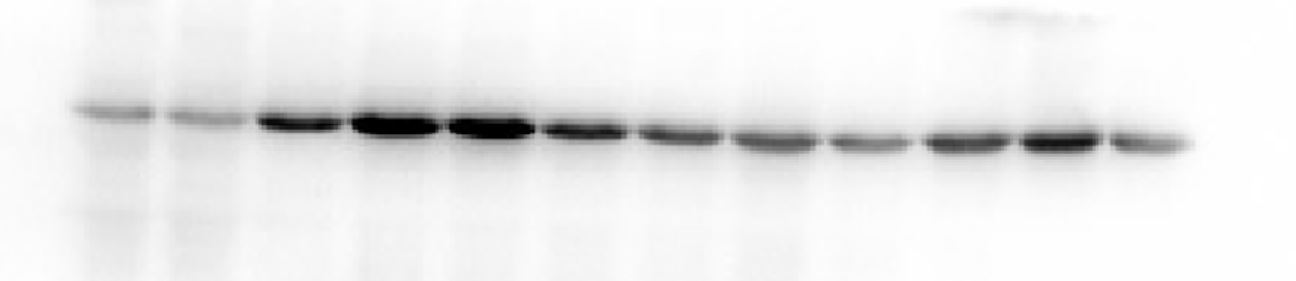

Supplement: Supplementary file 2 [file Data_Sheet_2.ZIP › wb/figure 4-a┬-actin(4).JPG]

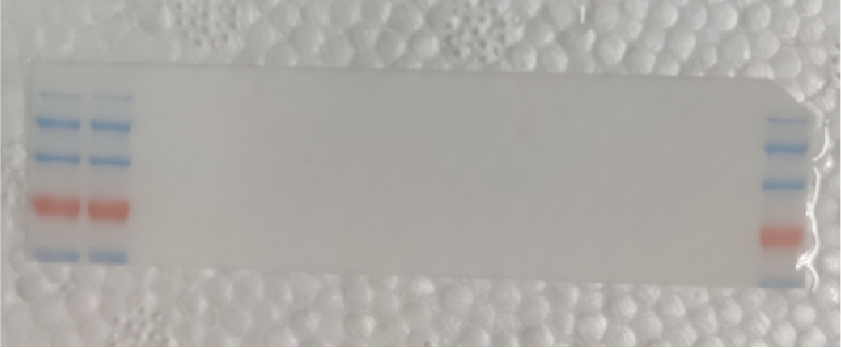

Supplement: Supplementary file 2 [file Data_Sheet_2.ZIP › wb/figure 4-a┬-catenin ú¿1ú⌐.png]

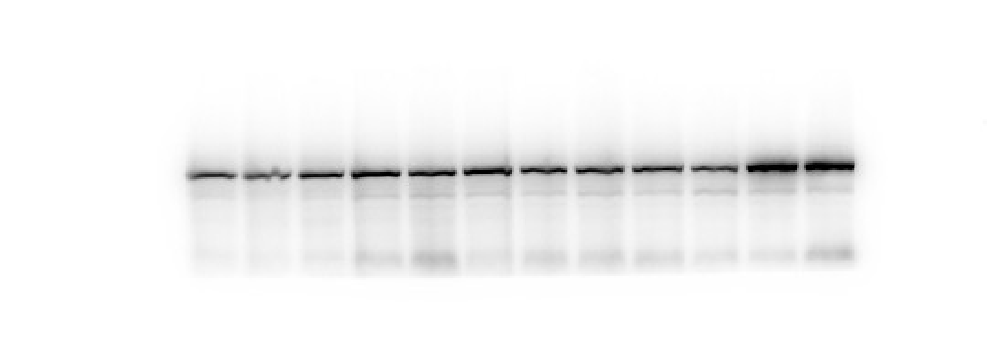

Supplement: Supplementary file 2 [file Data_Sheet_2.ZIP › wb/figure 4-a┬-catenin ú¿2ú⌐.png]

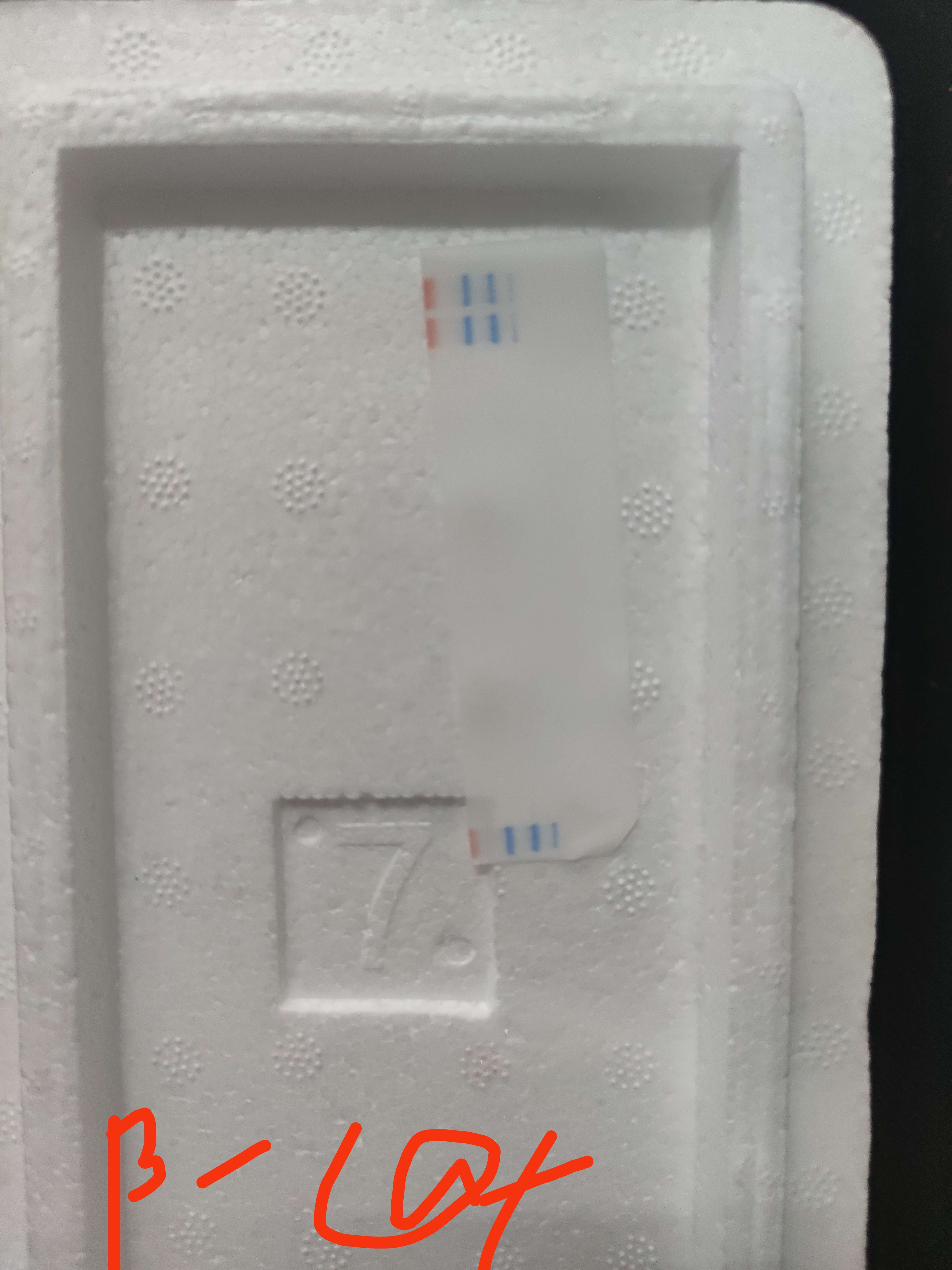

Supplement: Supplementary file 2 [file Data_Sheet_2.ZIP › wb/figure 4-a┬-catenin ú¿3ú⌐.jpg]

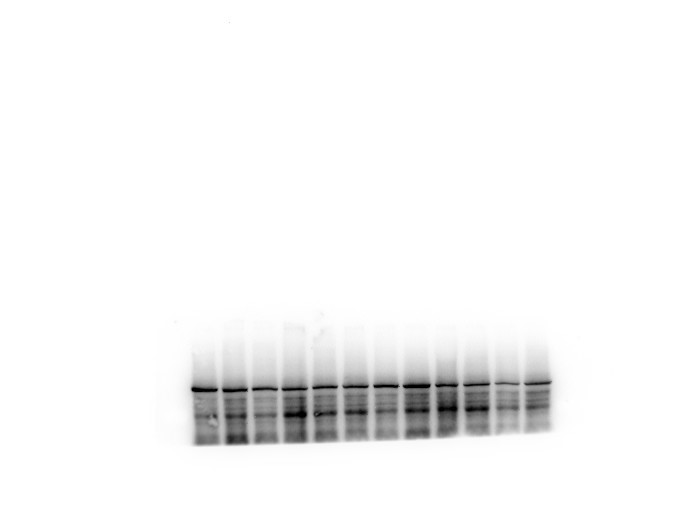

Supplement: Supplementary file 2 [file Data_Sheet_2.ZIP › wb/figure 4-a┬-catenin ú¿4ú⌐.jpg]

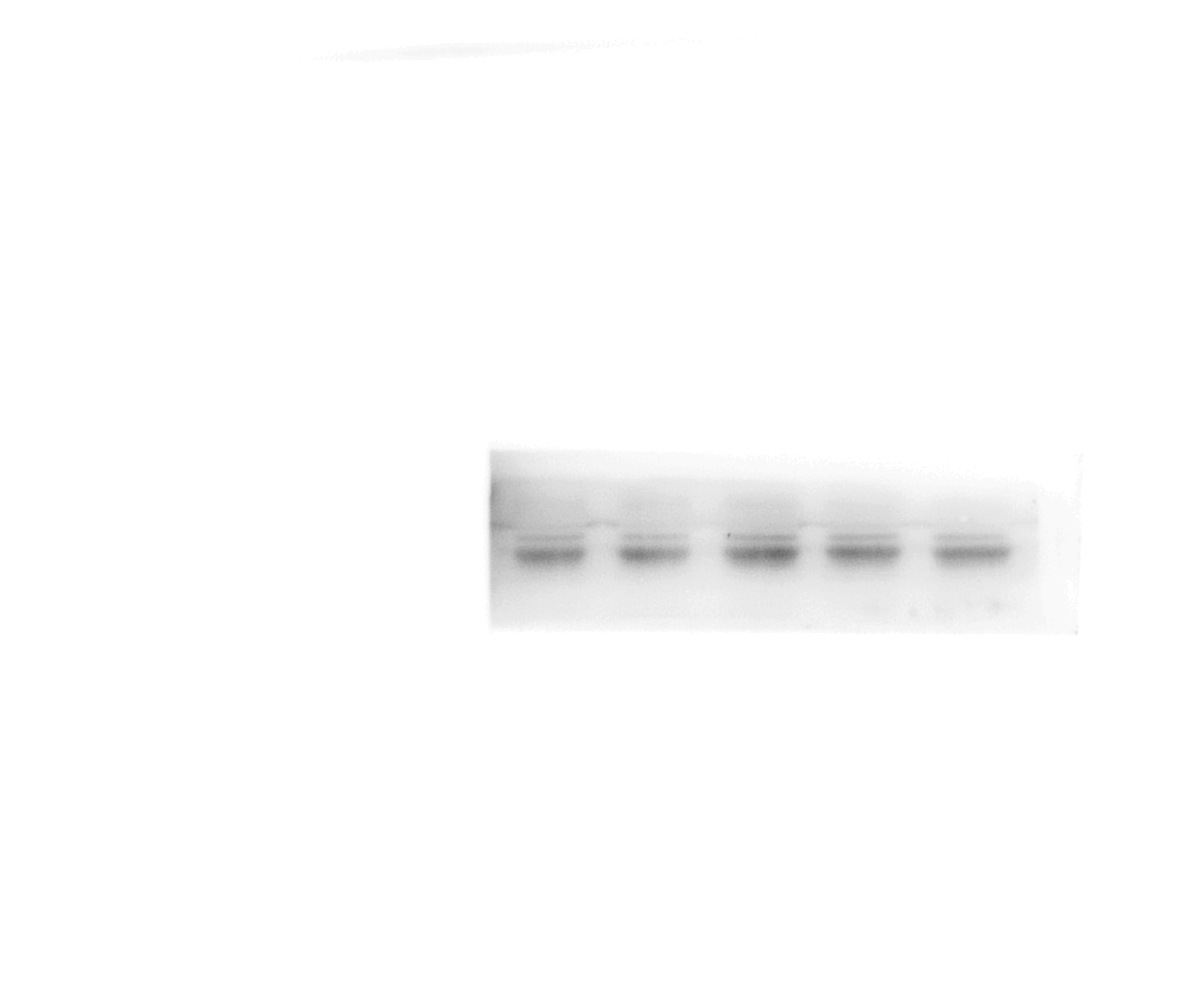

Supplement: Supplementary file 2 [file Data_Sheet_2.ZIP › wb/figure 5 wb/aSMA-16h.tif]

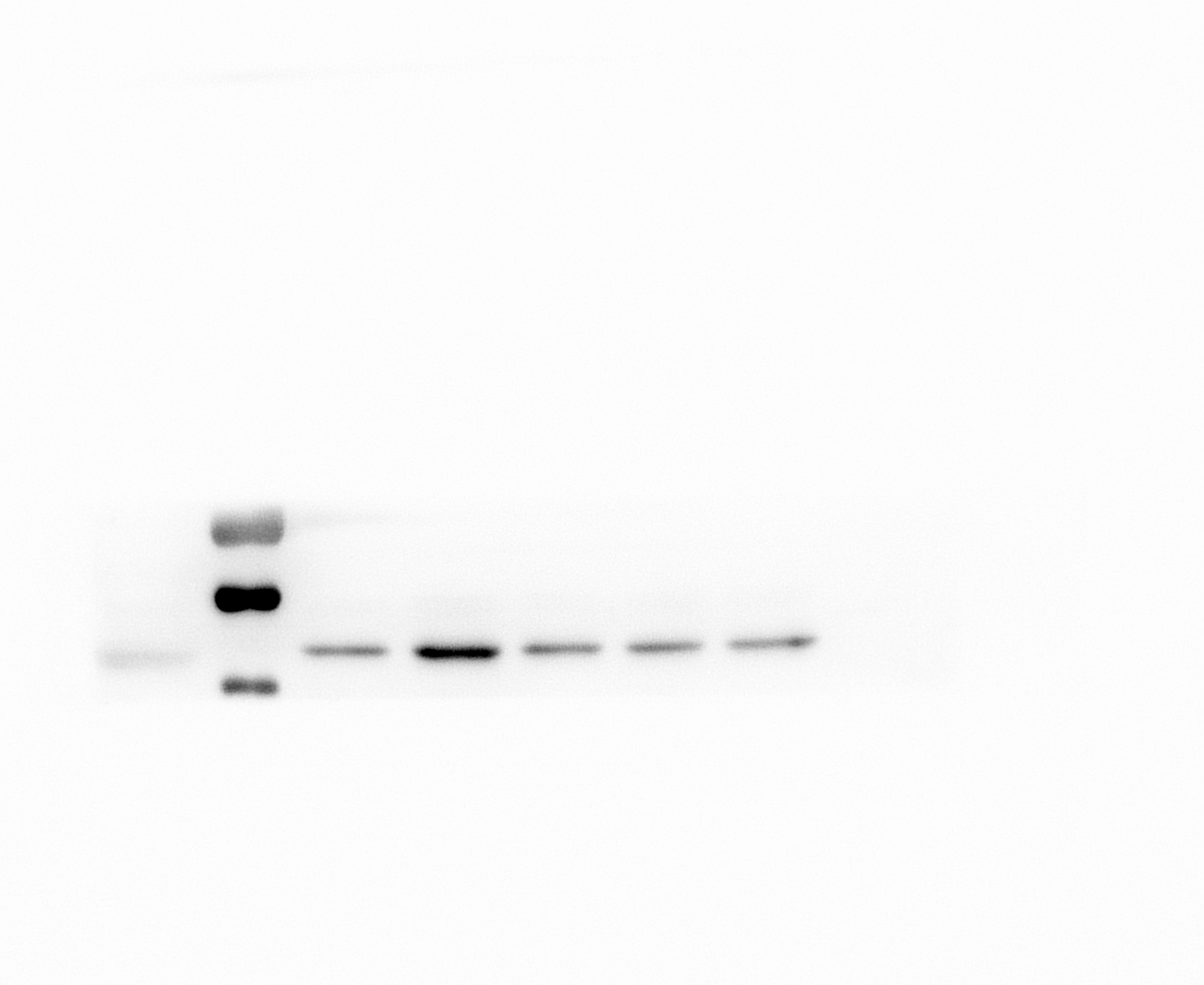

Supplement: Supplementary file 2 [file Data_Sheet_2.ZIP › wb/figure 5 wb/aSMA-24h.tif]

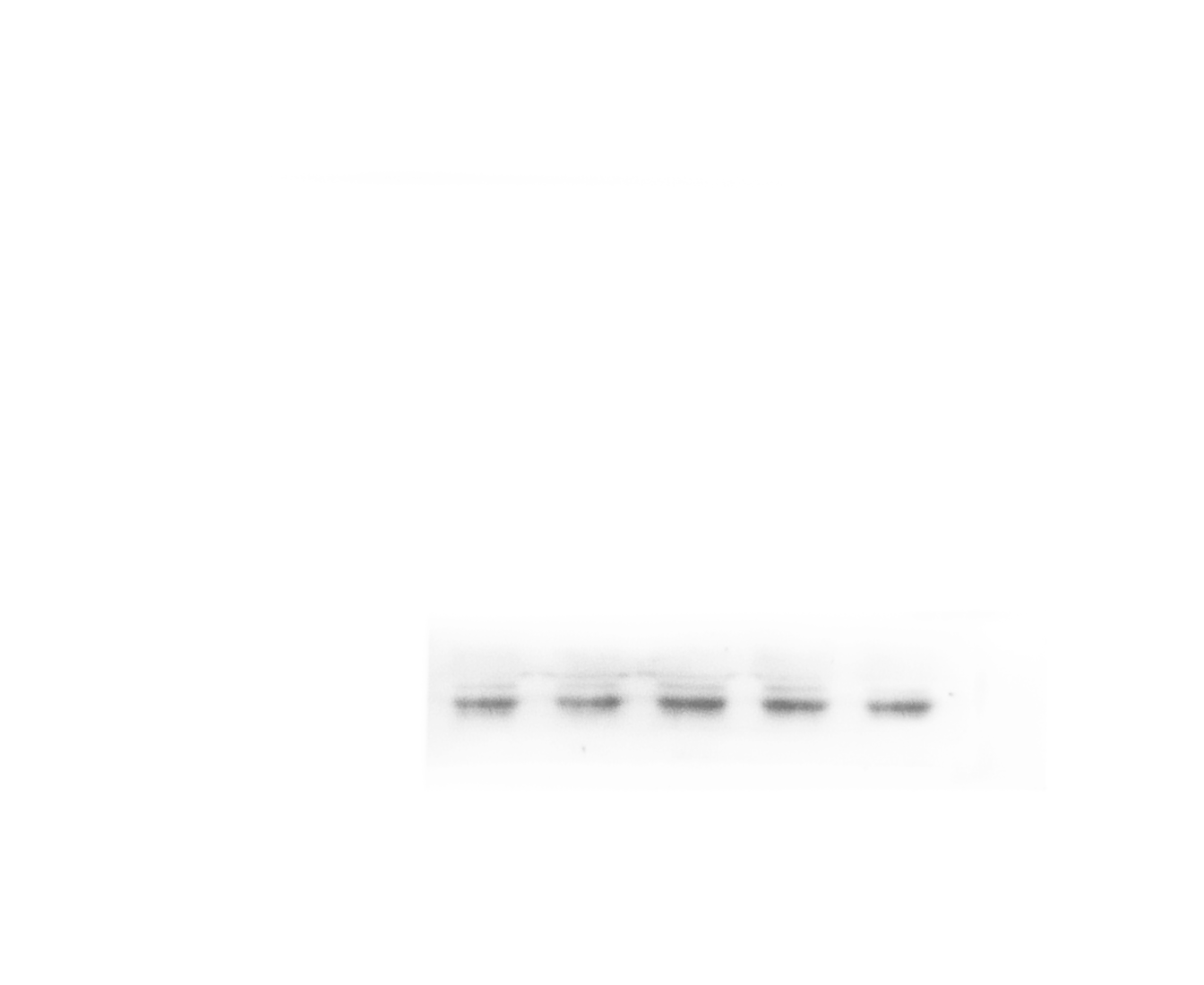

Supplement: Supplementary file 2 [file Data_Sheet_2.ZIP › wb/figure 5 wb/aSMA-36h.tif]

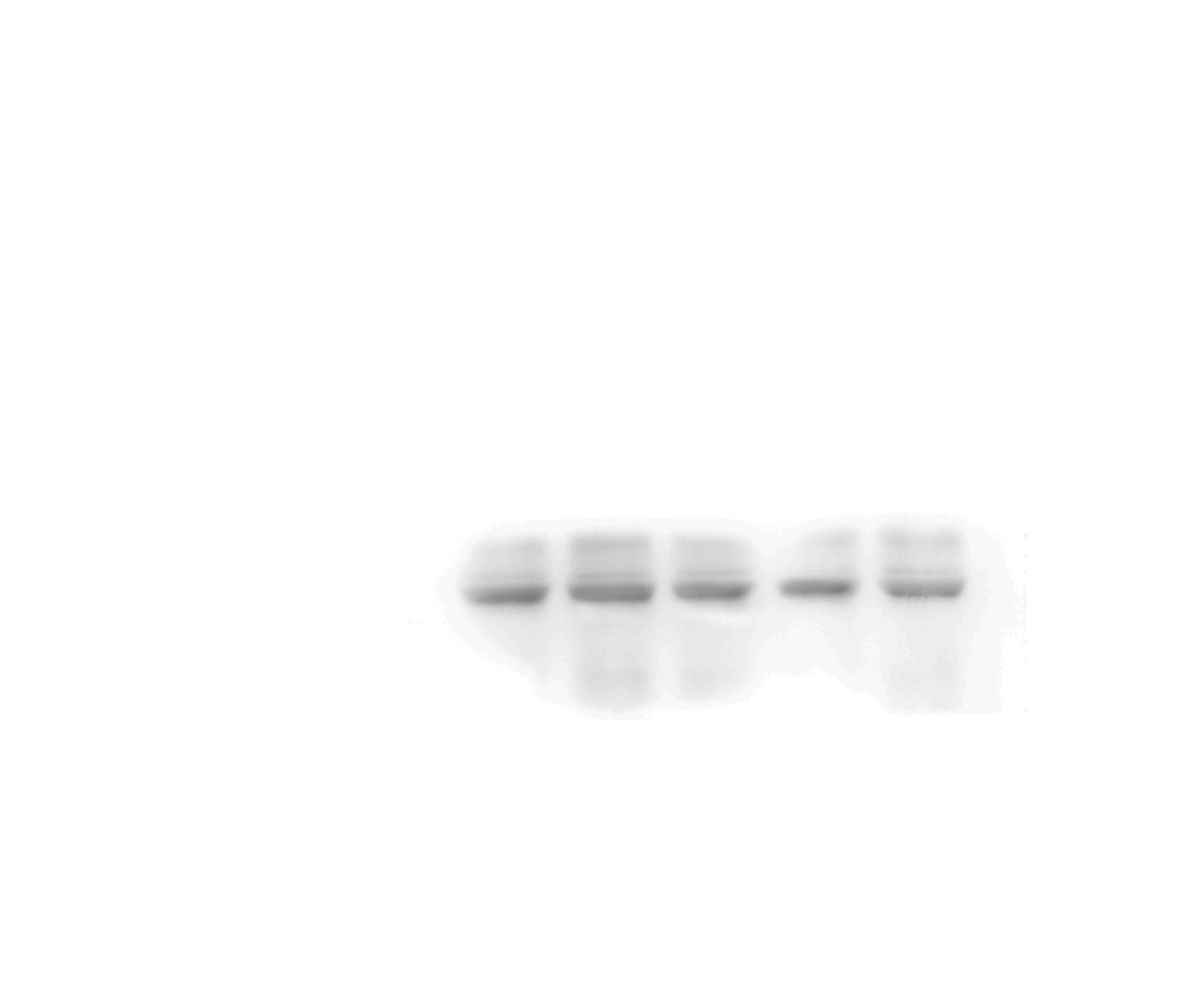

Supplement: Supplementary file 2 [file Data_Sheet_2.ZIP › wb/figure 5 wb/aSMA-6h.tif]

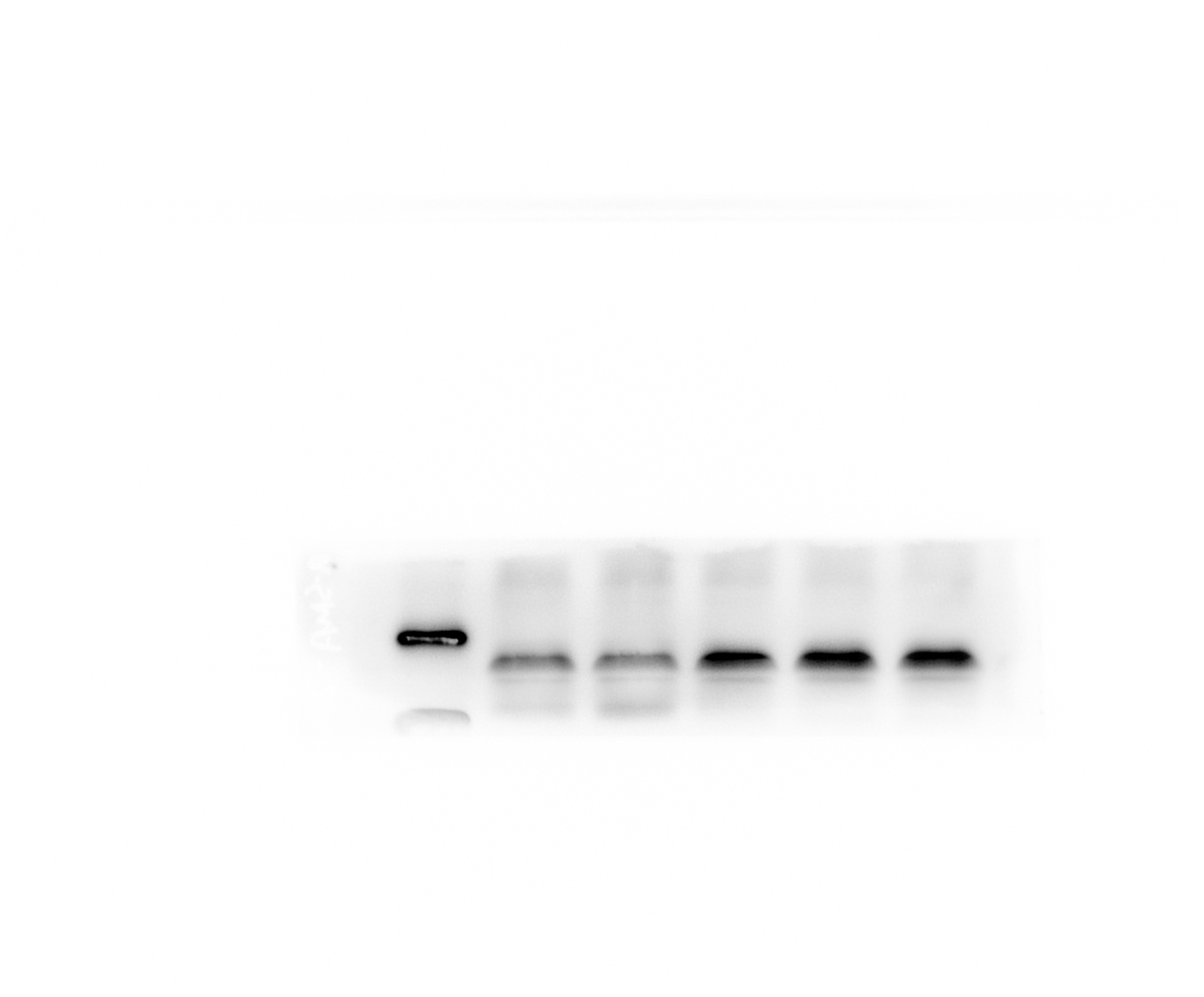

Supplement: Supplementary file 2 [file Data_Sheet_2.ZIP › wb/figure 5 wb/aSMA-72h.tif]

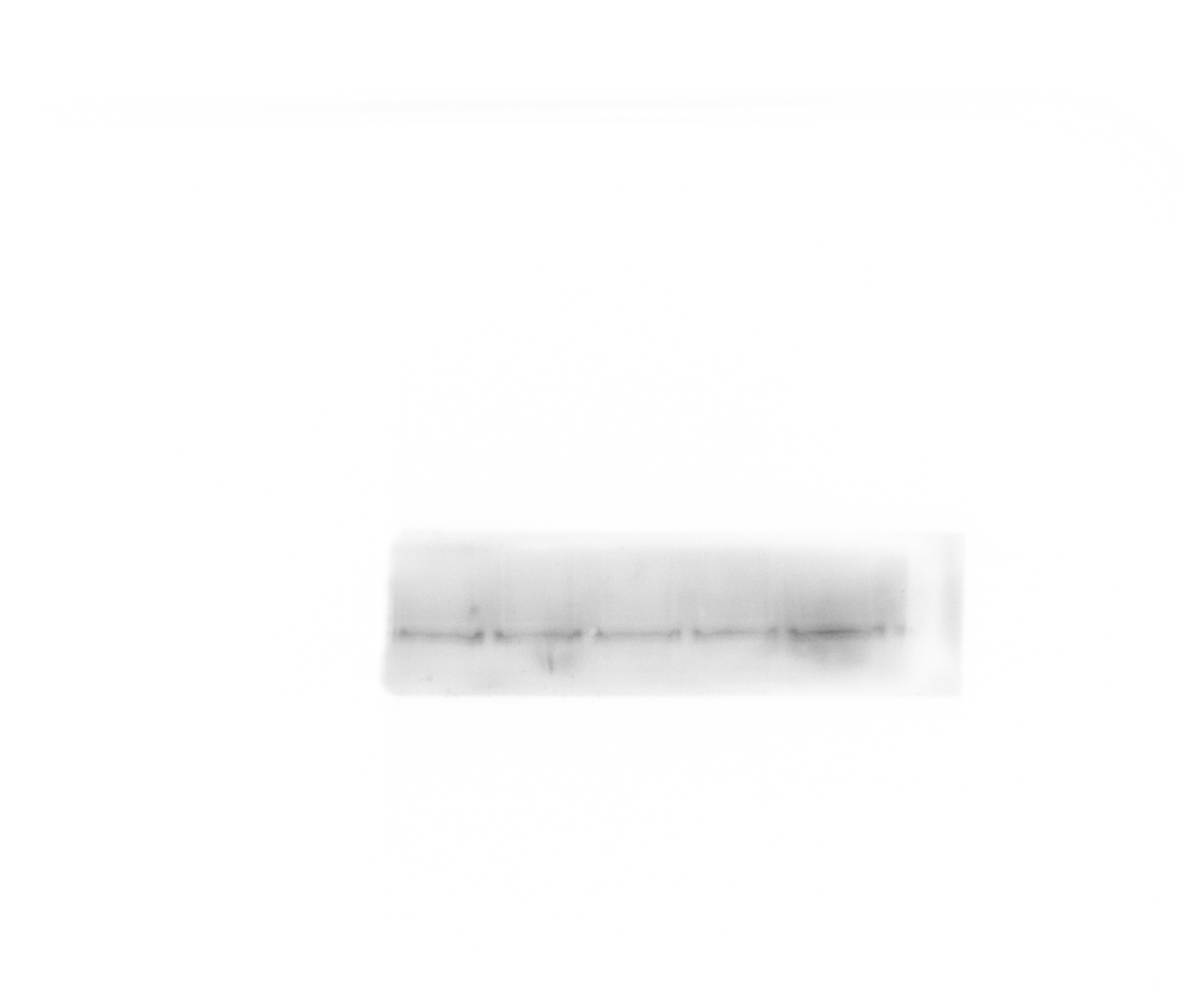

Supplement: Supplementary file 2 [file Data_Sheet_2.ZIP › wb/figure 5 wb/figure 5 COLLAGEN-16h.tif]

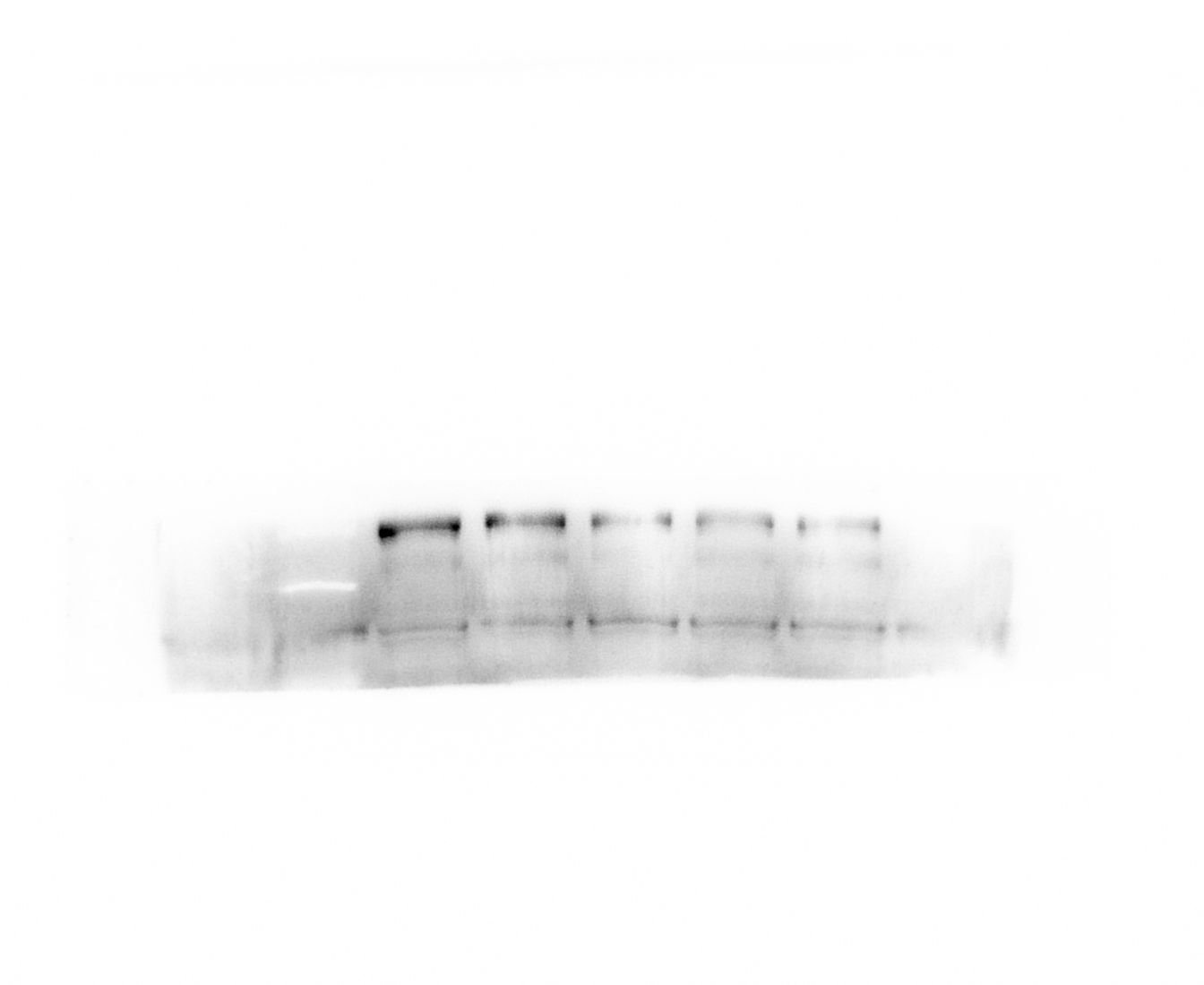

Supplement: Supplementary file 2 [file Data_Sheet_2.ZIP › wb/figure 5 wb/figure 5 COLLAGEN-36h.tif]

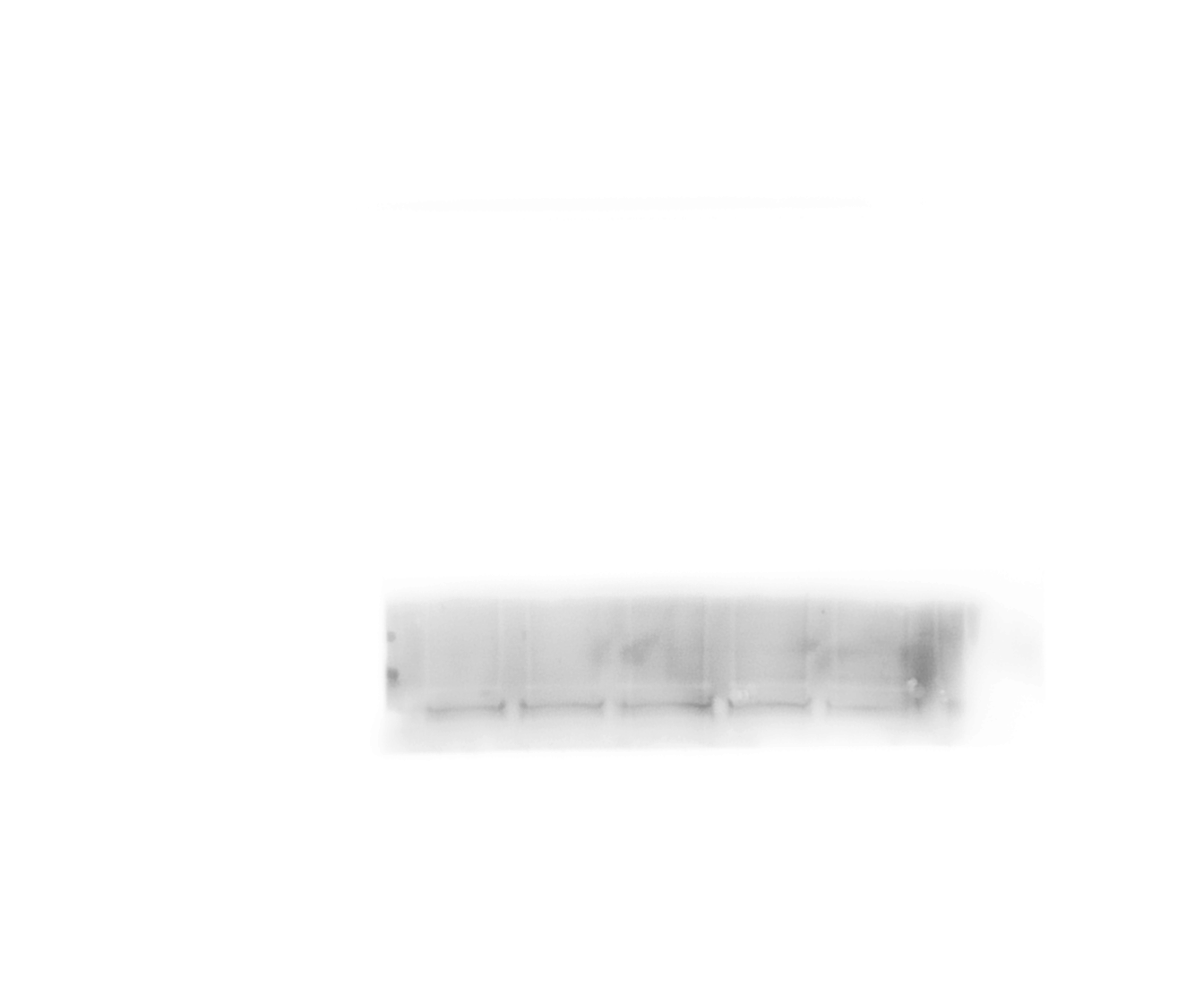

Supplement: Supplementary file 2 [file Data_Sheet_2.ZIP › wb/figure 5 wb/figure 5 COLLAGEN-6h.tif]

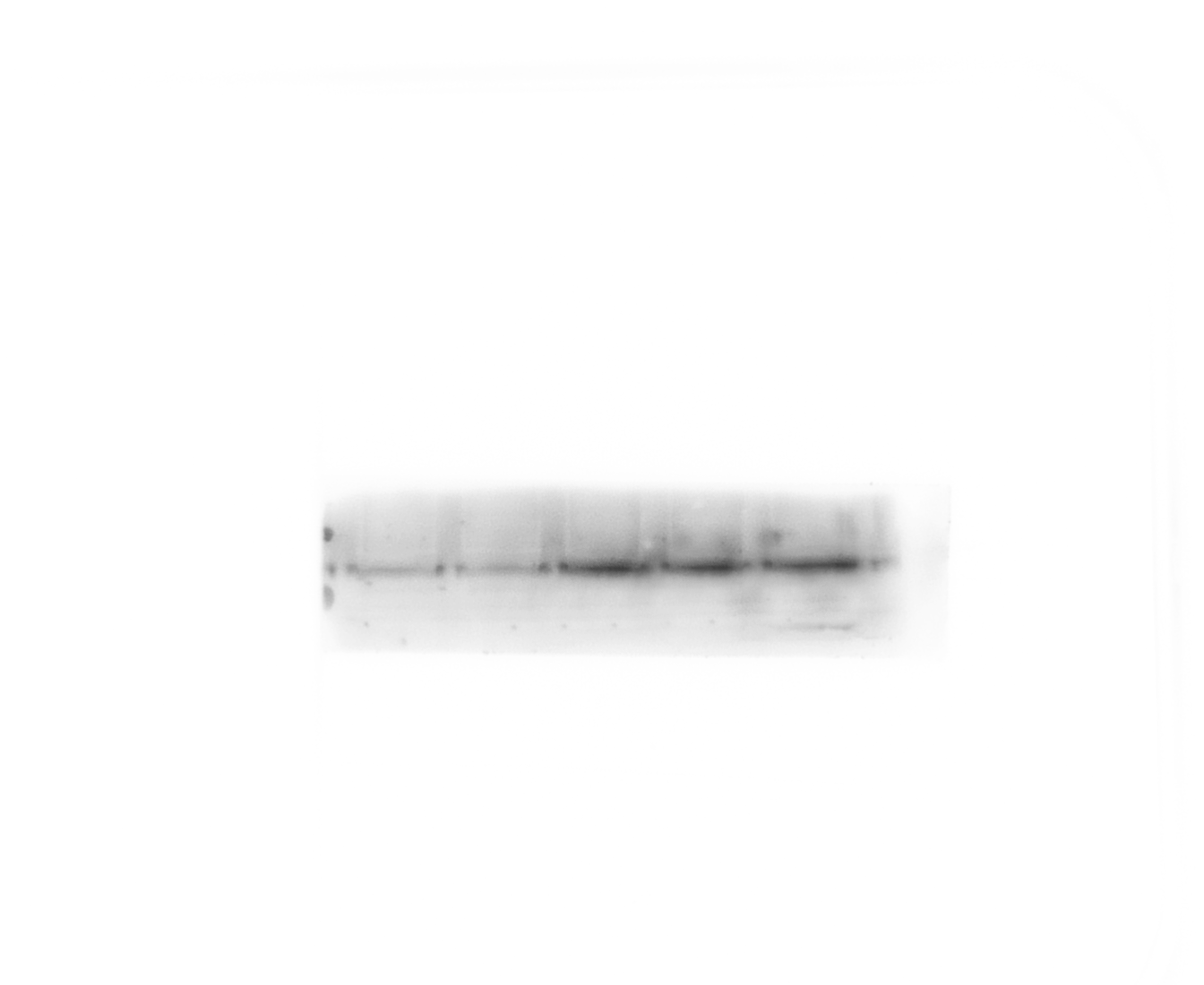

Supplement: Supplementary file 2 [file Data_Sheet_2.ZIP › wb/figure 5 wb/figure 5 COLLAGEN-72h.tif]

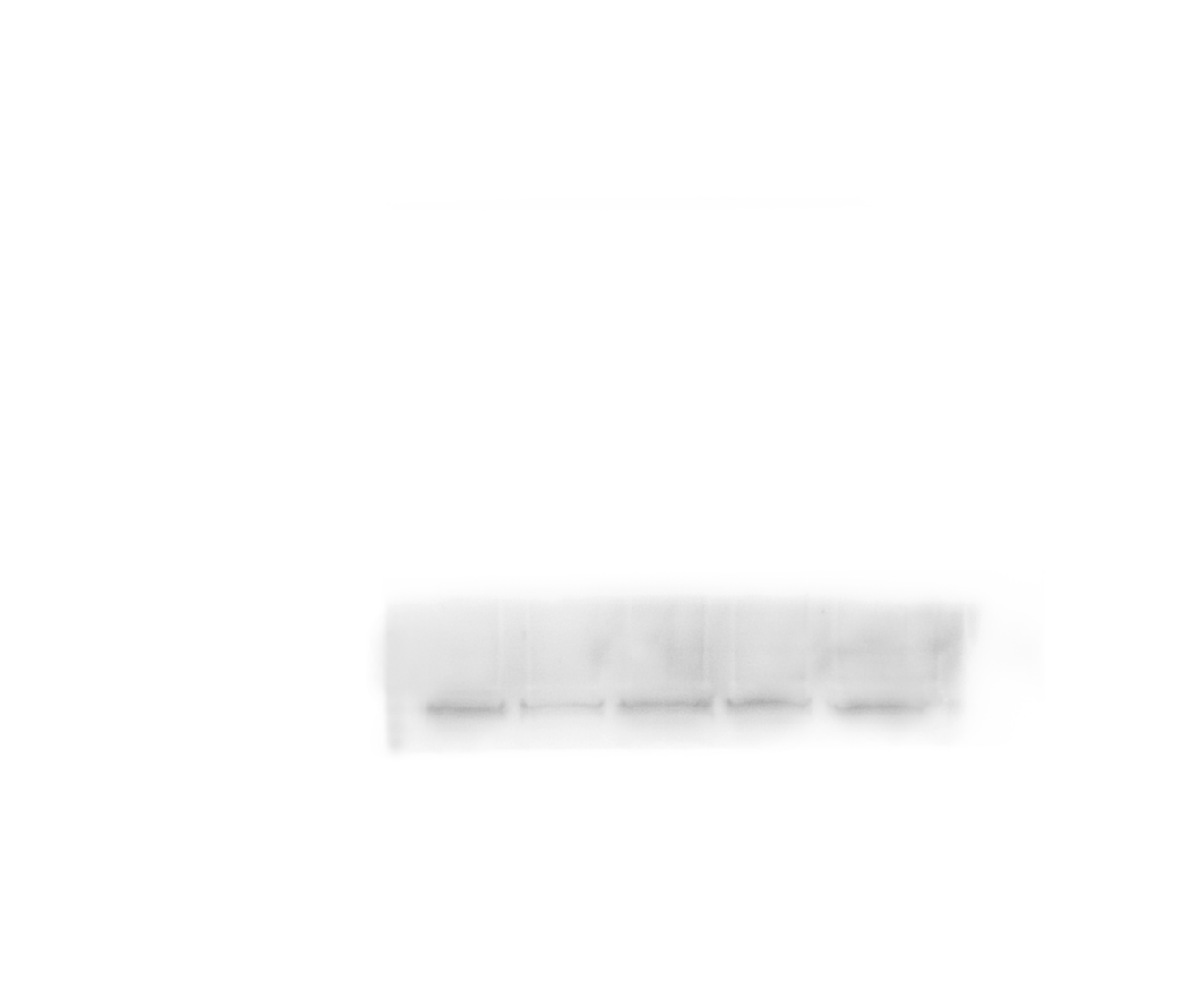

Supplement: Supplementary file 2 [file Data_Sheet_2.ZIP › wb/figure 5 wb/figure 5 COLLAGEN_24h.tif]

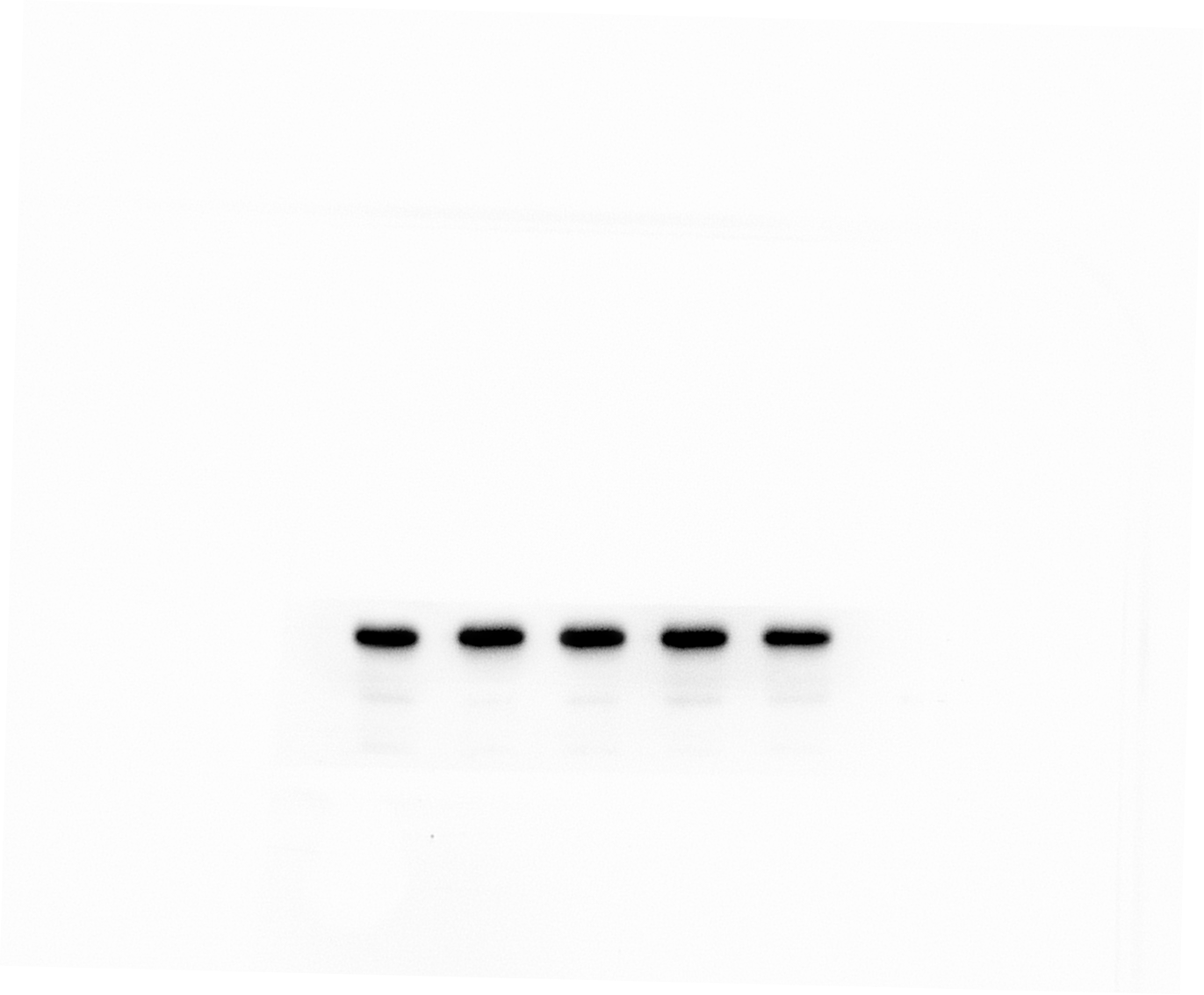

Supplement: Supplementary file 2 [file Data_Sheet_2.ZIP › wb/figure 5 wb/figure 5 gapdh-16h.tif]

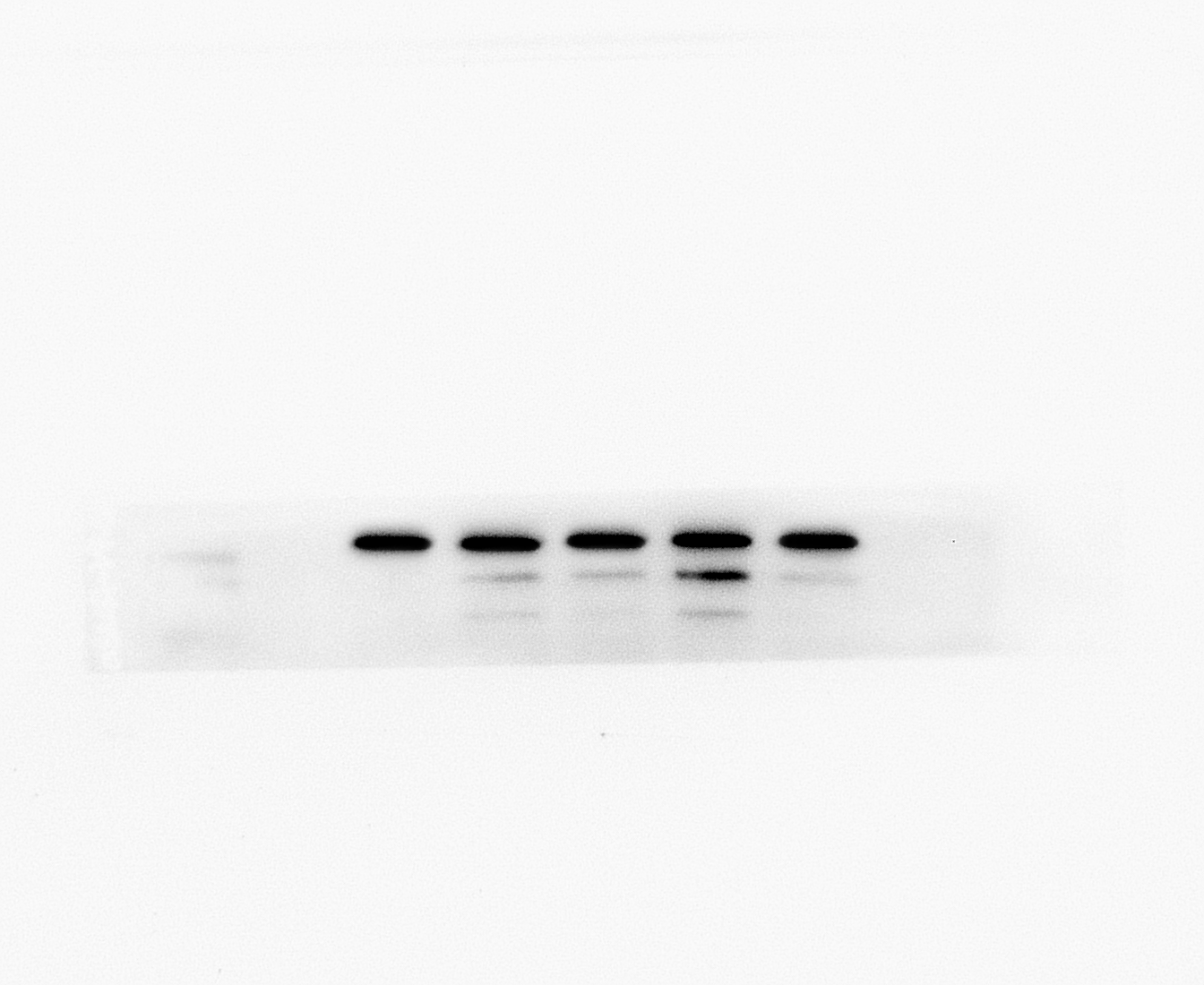

Supplement: Supplementary file 2 [file Data_Sheet_2.ZIP › wb/figure 5 wb/figure 5 gapdh-24h.tif]

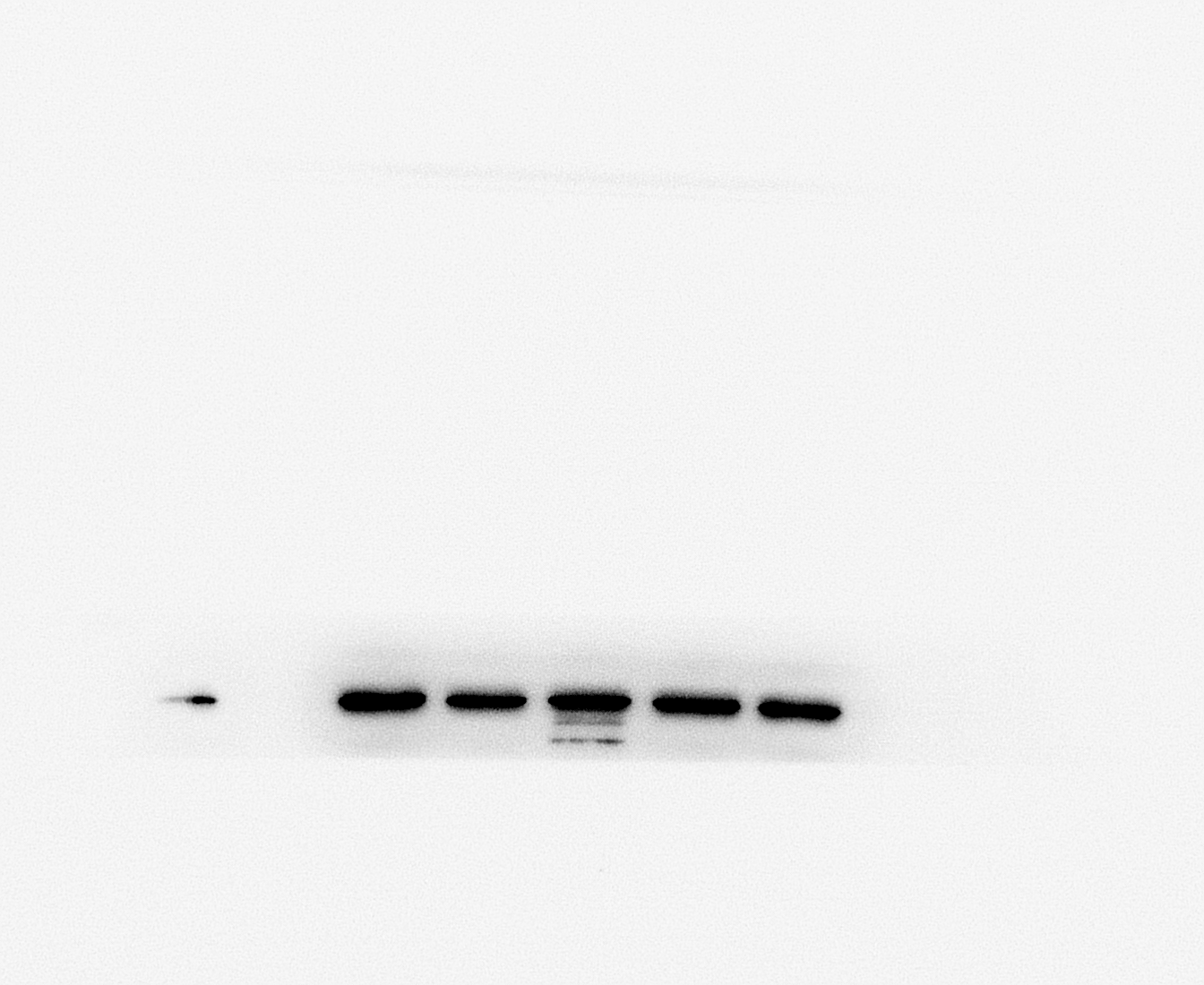

Supplement: Supplementary file 2 [file Data_Sheet_2.ZIP › wb/figure 5 wb/figure 5 gapdh-48h.tif]

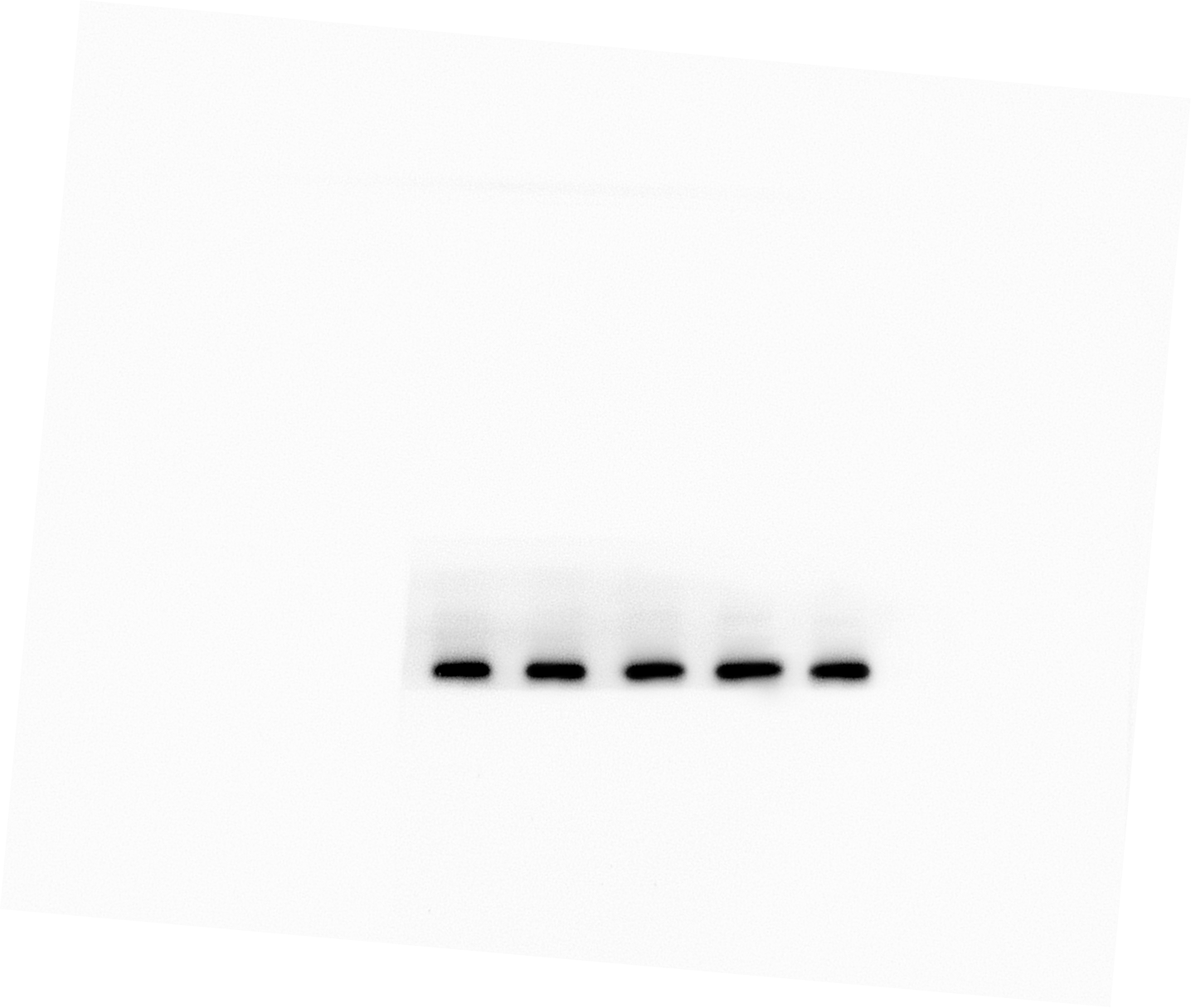

Supplement: Supplementary file 2 [file Data_Sheet_2.ZIP › wb/figure 5 wb/figure 5 gapdh-6h.tif]

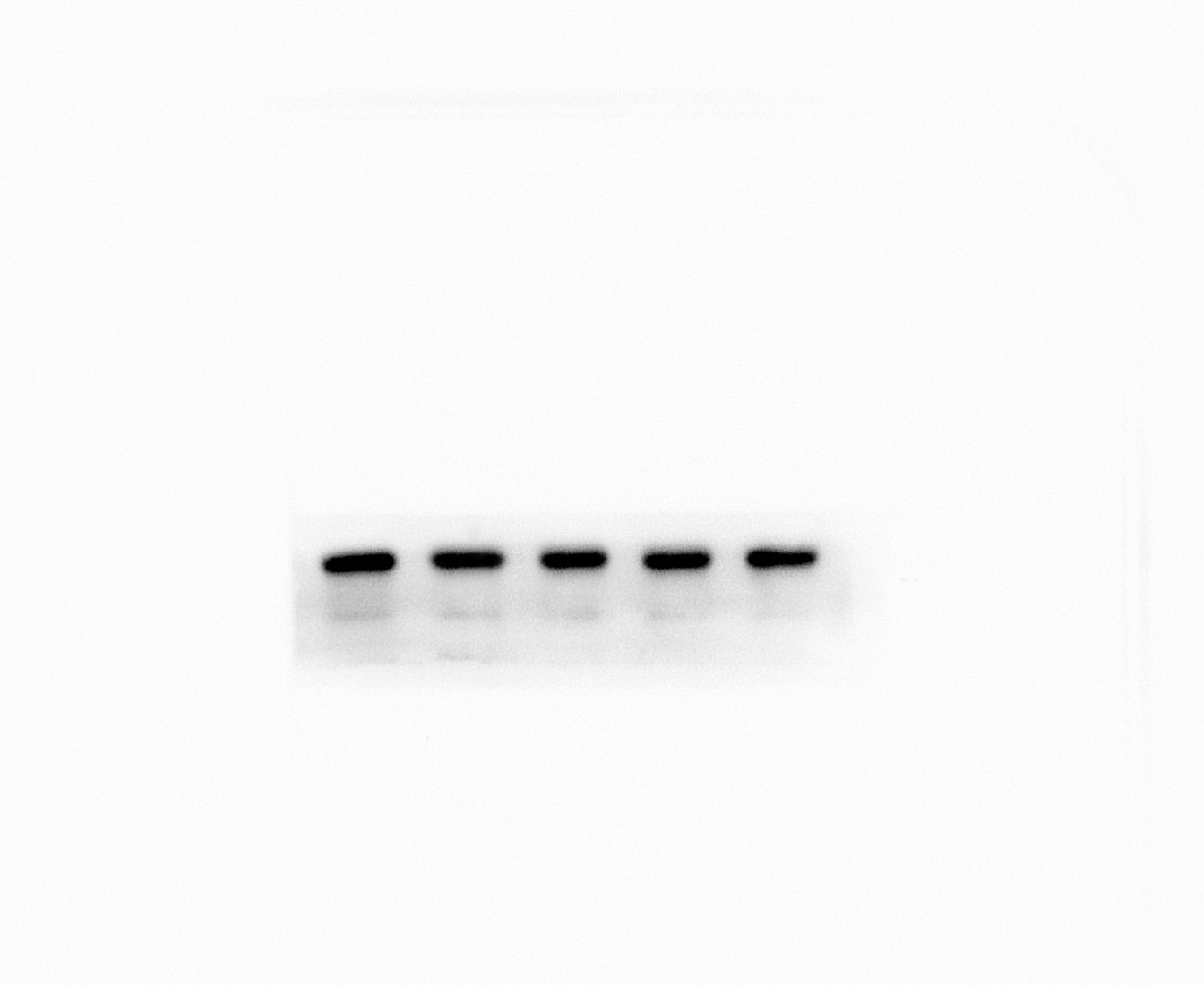

Supplement: Supplementary file 2 [file Data_Sheet_2.ZIP › wb/figure 5 wb/figure 5 gapdh-72h.tif]

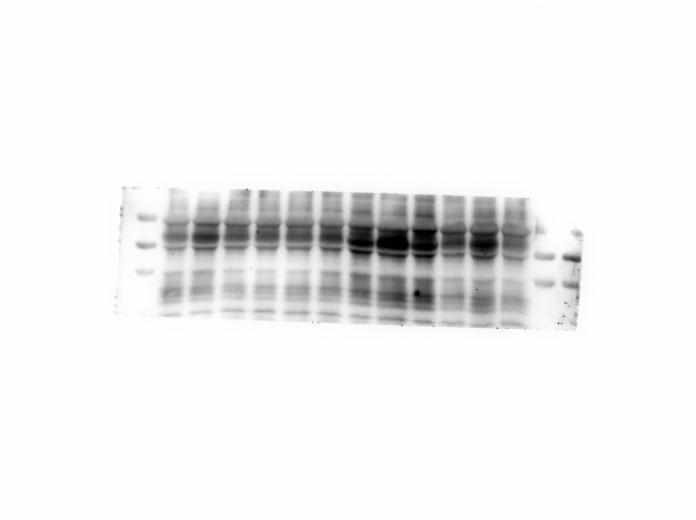

Supplement: Supplementary file 2 [file Data_Sheet_2.ZIP › wb/p-Smad23 1.jpg]

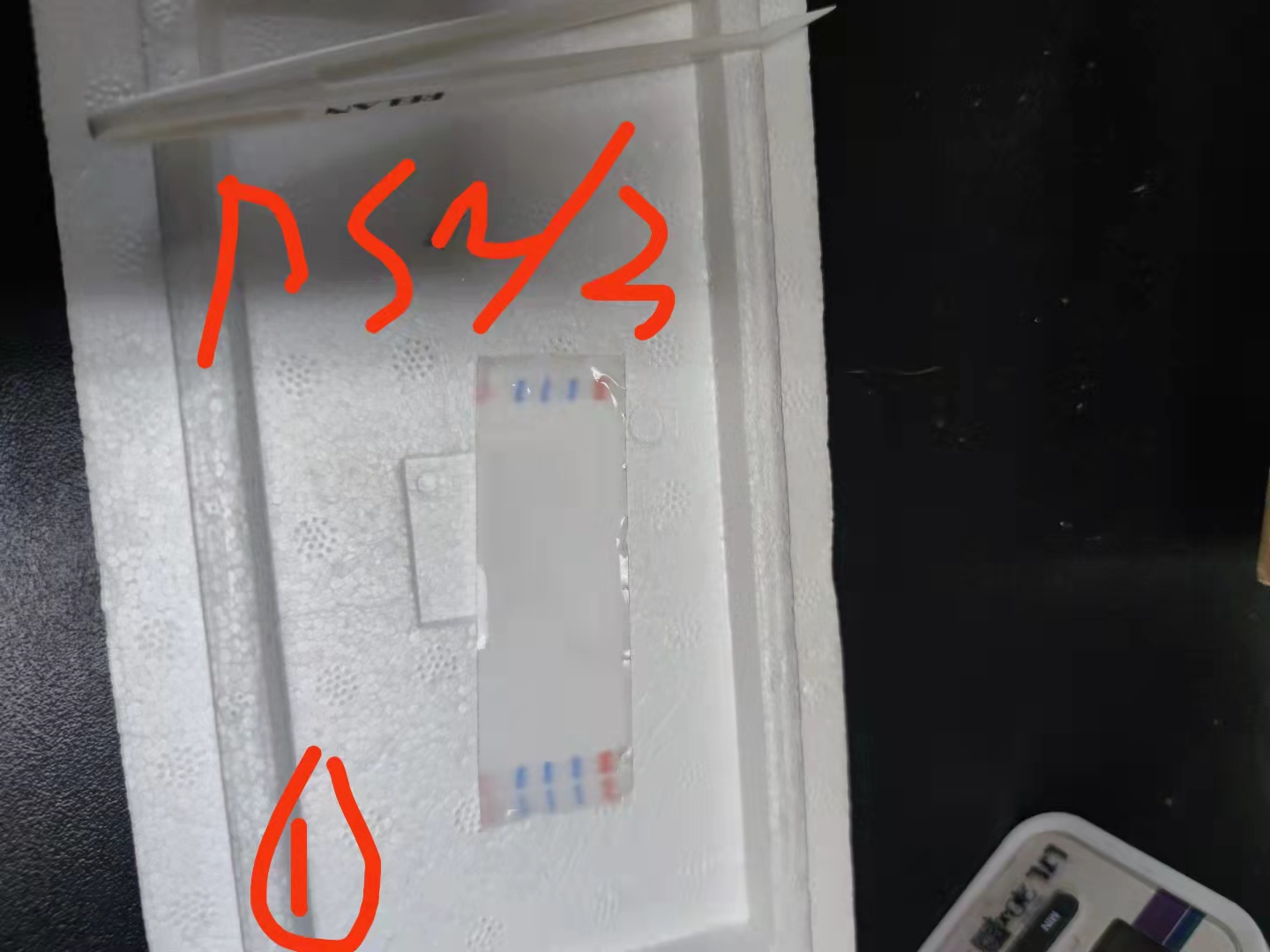

Supplement: Supplementary file 2 [file Data_Sheet_2.ZIP › wb/p-Smad23 2.jpg]

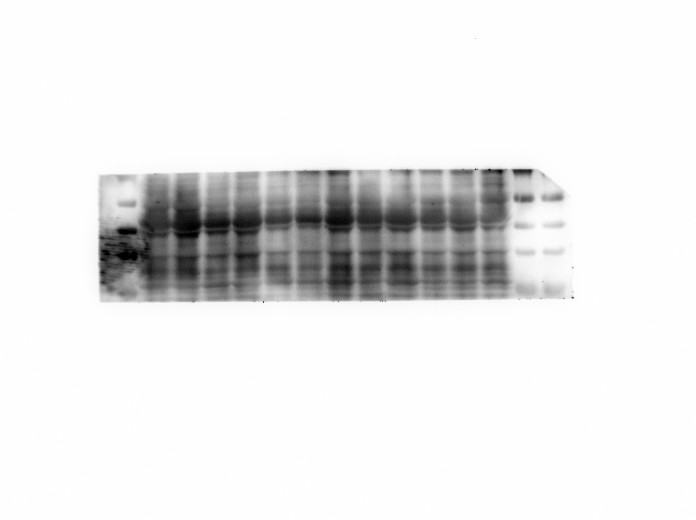

Supplement: Supplementary file 2 [file Data_Sheet_2.ZIP › wb/p-Smad23 3.jpg]

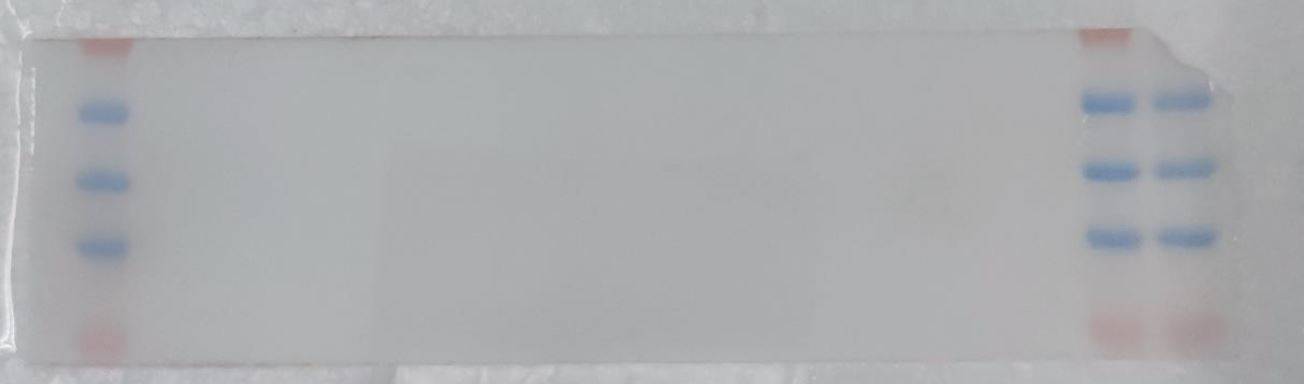

Supplement: Supplementary file 2 [file Data_Sheet_2.ZIP › wb/p-Smad23 4.jpg]
