# Supplementary material for: Tangshen Formula Improves Diabetes-Associated Myocardial Fibrosis by Inhibiting TGF-β/Smads and Wnt/β-Catenin Pathways
Source: Front Med (Lausanne). 2021 Dec 6;8:732042. doi: 10.3389/fmed.2021.732042 (PMC8687440; doi:10.3389/fmed.2021.732042)
Supplement: Supplementary file 1 [file Data_Sheet_1.ZIP › original data/microscope image.docx]

Dear editor:

The microscope image is too large to be uploaded to the submission link. I have uploaded the microscope picture to Jianguoyun. The file link is:

<https://www.jianguoyun.com/p/DdWa5H8Qhb3PCRjl1v0D.>

It is the first time to use the Jianguoyun,so please contact me if have any problems. My E-mail: dataohuhu@163.com.

Thank you for you review.

Sinscerely,

Lin Hu
